# Using the collaborative cross to identify the role of host genetics in defining the murine gut microbiome

**DOI:** 10.1186/s40168-023-01552-8

**Published:** 2023-07-08

**Authors:** Aravindh Nagarajan, Kristin Scoggin, Jyotsana Gupta, David W. Threadgill, Helene L. Andrews-Polymenis

**Affiliations:** 1 Interdisciplinary Program in Genetics, Texas A&M University, College Station, TX USA; 2 Department of Microbial Pathogenesis and Immunology, Texas A&M University, College Station, TX USA; 3 Department of Molecular and Cellular Medicine, Texas A&M University, College Station, TX USA; 4 Texas A&M Institute for Genome Sciences and Society, Texas A&M University, College Station, TX USA; 5 Department of Biochemistry & Biophysics and Department of Nutrition, Texas A&M University, College Station, TX USA

## Abstract

**Background:**

The human gut microbiota is a complex community comprised of trillions of bacteria and is critical for the digestion and absorption of nutrients. Bacterial communities of the intestinal microbiota influence the development of several conditions and diseases. We studied the effect of host genetics on gut microbial composition using Collaborative Cross (CC) mice. CC mice are a panel of mice that are genetically diverse across strains, but genetically identical within a given strain allowing repetition and deeper analysis than is possible with other collections of genetically diverse mice.

**Results:**

16S rRNA from the feces of 167 mice from 28 different CC strains was sequenced and analyzed using the Qiime2 pipeline. We observed a large variance in the bacterial composition across CC strains starting at the phylum level. Using bacterial composition data, we identified 17 significant Quantitative Trait Loci (QTL) linked to 14 genera on 9 different mouse chromosomes. Genes within these intervals were analyzed for significant association with pathways and the previously known human GWAS database using Enrichr analysis and Genecards database. Multiple host genes involved in obesity, glucose homeostasis, immunity, neurological diseases, and many other protein-coding genes located in these regions may play roles in determining the composition of the gut microbiota. A subset of these CC mice was infected with *Salmonella* Typhimurium. Using infection outcome data, an increase in abundance of genus *Lachnospiraceae* and decrease in genus *Parasutterella* correlated with positive health outcomes after infection. Machine learning classifiers accurately predicted the CC strain and the infection outcome using pre-infection bacterial composition data from the feces.

**Conclusion:**

Our study supports the hypothesis that multiple host genes influence the gut microbiome composition and homeostasis, and that certain organisms may influence health outcomes after *S.* Typhimurium infection.

Video Abstract

**Supplementary Information:**

The online version contains supplementary material available at 10.1186/s40168-023-01552-8.

## Introduction

The nutrient-rich environment of the human intestinal tract harbors up to 100 trillion microbes [1]. Sterile at birth, our colon becomes densely populated with microbes, in the range of 10^11^–10^12^ cells/ml, the highest recorded density for any microbial habitat [2]. These microbes become an integral part of the digestive system breaking down complex molecules, modifying host-derived molecules (like bile acids for example), and modulating the immune response [3].

Differential abundance of certain microbes in the intestine has been implicated in various diseases, including inflammatory bowel disease (IBD), obesity, and food allergies [4–6]. Some microbial metabolites interact with the gut-brain axis and have been associated with autism [7], Parkinson’s disease [8], Alzheimer’s disease [9], epilepsy [10], and stroke [11]. Other microbes influence circadian rhythm disturbances, depression, and disruption of sleep patterns [12]. Despite these associations, establishing causal relationships between a microbiome and a disease is challenging.

The recombinant inbred panel of mice called the Collaborative Cross (CC) was created to model a genetically diverse population. Eight founder strains, A/J, C57BL/6 J, 129S1/SvImJ, NOD/LtJ, NZO/HiLtJ, CAST/Ei, PWK/PhJ, and WSB/EiJ, were bred in a funnel fashion to generate a large panel of inbred strains with balanced allele frequency and dense, evenly distributed recombination sites. The CC panel approximates the genetic diversity found in humans and has a high resolution for mapping quantitative trait loci (QTL) [13–15]. CC mice have been widely used to define host genetic loci implicated in various infectious diseases including West Nile virus, tuberculosis, and influenza as well as other phenotypes including glucose tolerance, DNA damage, and Epilepsy [16–22]. In Ebola infections in CC mice, a diverse phenotypic variation ranging from high resistance to complete lethality was observed across CC strains, and a central transcriptional regulatory gene called *Tek* (TEK receptor tyrosine kinase) correlated with weight loss and mortality after infection [17]. In CC mice, liver and spleen bacterial load varies across strains after infection with *S*. Typhimurium (STm) [23]. A candidate gene *Slc35f1* (solute carrier family 35, member F1), which has lactate dehydrogenase activity and is predicted to influence the pyruvate metabolism pathway in STm, was identified as potentially important [24].

Though the microbiome has traditionally been seen as influenced by the environment, recent studies have linked the influence of host genetics to microbiome composition [25]. In human twin studies, bacterial representation was more similar in monozygotic twins than in dizygotic twins, supporting a positive association between genetic factors and microbiome [26]. Re-analysis of data from previous human twin studies has established a similar association with the host genetics and the microbiome [26–28]. The effects of genetics, diet, and the environment make establishing causal relationships challenging in human studies.

In murine models with controlled diet and environment, a relationship between the host genetic makeup and the microbiome is becoming apparent. QTL analysis on the advanced intercross mouse population has shown that microbial abundance is a polygenic trait [29, 30]. Pleiotropy in QTL regions was observed for both closely related and unrelated bacteria. Other mouse population analyses identified several microbial abundance QTLs and correlated them to immune response, obesity, and insulin secretion genes from the host [31, 32]. The CC population has also been used to associate microbial metabolites with GI cancers, lipid metabolism, and inflammation [33]. Recently, the intestinal microbiome composition of CC mice has been associated with a sleep phenotype, memory, anxiety-like behavior, and Azoxymethane-induced toxicity [34–37].

Over a 2-year time period, we collected feces from 28 CC mouse strains, including three males and three females of each strain, and performed 16s rRNA gene sequencing. Individuals within a strain had similar microbiota composition, while there were significant differences between the strains starting at the phylum level. Microbial abundance data at the genus level identified significant genes associated with individual genera across the genome. After collection of feces, a subset of CC strains was infected with *Salmonella enterica* serotype Typhimurium (STm) in a parallel series of experiments [23]. We used the survival data after STm infection to identify and correlate the microbes associated with STm infection outcome. A machine learning algorithm predicted the correct CC strain and the infection outcome using bacterial composition data. The increase in abundance of genus *Lachnospiraceae* and decrease in genus *Parasutterella* correlated with a positive health outcome after infection.

## Methods

### Animals

Eight- to 12-week-old male and female Collaborative Cross mice (CC) were bred at the Division of Comparative Medicine at Texas A&M University. In preparation for a different series of experiments, mice were implanted with an E-mitter telemetry device (STARR Life Sciences Corp.) and permitted to recover for 1 week (detailed methodology described in [23]). All mice were transferred to a second facility and individually housed at least 5 days before the collection of feces. Individually housed mice had hardwood chip bedding in ventilated cages, with nestlet squares, a cardboard hut, and were fed a standardized rodent diet (Teklad Global 19% protein extruded rodent diet, irradiated, 2919, from ENVIGO) and sterile water ad libitum.

### Ethics statement

Mouse studies were conducted in accordance with the Guide for the Care and Use of Laboratory Animals of the National Institutes of Health. All mouse studies were conducted at the Texas A&M Health Science Center using protocols (AUP—2015–0315D and 2018–0488D) approved by the Texas A&M Institutional Animal Care and Use Committee (IACUC).

### Fecal collection, storage, and DNA extraction

Feces were collected by placing mice on a paper towel under an inverted glass beaker. Sterile forceps were used to transfer at least three fecal pellets per animal to cryovials and feces were stored at − 80 °C until use. Total DNA from one fecal pellet for each animal was extracted using MagAttract PowerSoil DNA EP Kit from Qiagen.

### *Salmonella* infection

CC mice were infected by gavage with virulent wild-type *Salmonella enterica* serotype Typhimurium strain HA420 (ATCC14028s nal^r^) as previously described [23, 38]. Briefly, HA420 was grown to the stationary phase at 37 °C with aeration, serially diluted, and plated for bacterial CFU to determine the exact titer of the inoculum. One week after moving to a different facility, mice were inoculated by gavage with approximately 2–5 × 10^7^ CFU of *S.* Typhimurium HA420 in 100 µl of LB broth. Body temperature and activity data from the telemetry device, body condition scores, and weight loss data from daily health checks were used to monitor disease progression. Mice that developed severe clinical symptoms were humanely euthanized by CO_2_ asphyxiation. If mice remained healthy at the end of the experiment, they were euthanized at 7 days post-infection.

### Sequencing of 16S rRNA genes

Amplicon libraries were prepared for the variable V3 and V4 regions of the prokaryotic 16S rRNA gene. Briefly, extracted microbial DNA from one fecal pellet per animal was amplified using 16S amplicon PCR forward primer = 5′ TCGTCGGCAGCGTCAGATGTGTATAAGAGACAGCCT.

ACGGGNGGCWGCAG and 16S amplicon PCR reverse primer = 5′ GTCTCGTGGGCTCGGA.

GATGTGTATAAGAGACAGGACTACHVGGGTATCTAATCC in a BIORAD thermocycler using the following cycling conditions (denaturation at 95 °C for 3 min followed by 25 cycles of 95 °C for 30 s, 55 °C for 30 s, 72 °C for 30 s, and a final extension at 72 °C for 5 min). The PCR products were purified with AMPure XP beads to purify the V3 and V4 amplicon to be free from primers and primer-dimer species. The purified amplicons were dual indexed using a Nextera XT Index kit and sequenced using the Illumina Miseq platform at the Texas A&M Institute of Genome Sciences and Society (TIGSS) to generate 2 x300 base pair (bp) paired-end sequences.

### Preprocessing of sequences

A DADA2 pipeline was used for processing the 16S Illumina amplicon sequence data. Forward reads were trimmed at 17 and 285 bp and reserve reads were trimmed at 21 and 205 bp to remove the primers and bases with a median quality score of less than 25. Denoising, merging, and removal of chimeric sequences reduced the total sequences from 31,405,204 to 11,346,536 (Fig S1). After removing the Amplicon Sequence Variants (ASVs) that were not present in at least two samples, and mitochondrial and chloroplast sequences, 1637 unique ASVs were obtained.

### Data processing

Metagenomic bioinformatic analysis was performed using QIIME 2 2020.6 [39]. Raw sequence data were demultiplexed and quality filtered using the q2-demux plugin followed by denoising with DADA2 (via q2-dada2) [40]. Taxonomy was assigned to Amplicon Sequence Variants (ASVs) using the q2-feature-classifier [41] classify-sklearn naïve Bayes taxonomy classifier against Silva 138 SSURef NR99 full-length OTU reference sequences specifically trained using the 16S rRNA gene sequencing primers at 99% identity [42]. The feature table was filtered to include only the assigned reads of the kingdom bacteria and to remove singleton features and mitochondrial and chloroplast sequences. All amplicon sequence variants (ASVs) were aligned with mafft [43] (via q2-alignment) and used to construct a phylogeny with fasttree2 [44] (via q2-phylogeny). Samples were rarefied (subsampled without replacement) to 21,250 sequences per sample to reduce the effect of sampling depth. Alpha-diversity metric (Shannon’s Index), beta diversity metrics (unweighted UniFrac [45], and Bray–Curtis dissimilarity [46]), and principal coordinate analysis (PCoA) were estimated using q2-diversity. Group significance between alpha and beta diversity indexes were calculated with QIIME2 plugins using the Kruskal–Wallis test and permutational multivariate analysis of variance (PERMANOVA), respectively [47]. Additionally, analysis of similarity (ANOSIM) was performed on the beta diversity values [48]. Specific cage information for these animals was lost during a system shift in our animal facility. Therefore, we used the date of animal arrival to our facility over a 2-year period, combined with strain to calculate littermate effects.

### ANCOM

16S analysis of a single hypervariable region does not provide accurate classification at the species level. Thus, we used genus-level data. The abundance table was reduced to genus level and taxa that were not present in at least four different CC strains were removed. This table was used for further downstream analysis. Differential abundance testing was performed using ANCOM, to compare the relative abundance of a taxon between two groups by performing statistical tests on data transformed by an additive log-ratio of the abundance of a given taxon versus the abundance of all other taxa individually [49]. The analysis was performed using the R package—ANCOMBC, considering structural zeros grouped by strain and sex as covariates [50].

### Heritability

Broad-sense heritability was calculated using the formula *H*^2^ = *V*_G_/*V*_P_ = *V*_G_/(*V*_E_ + *V*_G_) as previously described [51, 52]. *V*_G_ and *V*_E_ are the variance explained by the genetic and the environmental component respectively while *V*_P_ is the total phenotypic variance for a given trait. Genus-level abundance data was used for this analysis. For each phenotype, *V*_G_ was calculated as the mean variance between the replicates of the same strain. *V*_P_ was calculated as the variance across all the strains for each corresponding phenotype. The maximum value for heritability is one.

### QTL analysis

QTL analysis was performed using R/qtl2 software [53]. This method performs genome scans using a linear mixed model to account for the complex population structure in the CC mice. Rank-transformed genus-level abundance was used as the phenotype and the genotypes were imputed from QTLViewer [54]. A genome-wide scan was performed using the scan1 function. The generated Logarithm of Odds (LOD) score is the likelihood ratio comparing the hypothesis of a QTL at a given position versus that of no QTL. To establish genome-wide significance, each individual phenotype was randomly shuffled 999 times and LOD scores were calculated for each iteration. Values at or above the 85^th^ percentile were considered significant for that phenotype.

### Candidate gene selection and enrichment

The Genomic confidence interval was calculated by dropping the LOD scores by 1.8, for each significant peak. The Mouse Genome Informatics (MGI) was used to find genes and QTL features within each interval [55]. To further shortlist candidate genes, the founder strain distribution pattern was queried against the CC variant database (V3 version) [56]. Variants were further shortlisted based on the impact scores calculated by the Ensembl Variant Effect Predictor (VEP) [57]. GeneCards database was used to collect human gene summaries, previously known human GWAS and Super pathways [58]. Gene enrichment analysis was performed using Enrichr [59, 60]. Briefly, genes with variants that matched the founder strain distribution pattern were imputed in the Enrichr web interface. Data tables for KEGG Human pathways 2021 and MGI mammalian phenotype 2021 were downloaded. Terms with a *P*-value of < 0.05 were considered significant.

### Machine learning

A random forest classifier (scikit-learn 0.24.2) was used to perform the machine learning analysis on the genus-level data [61]. For the strain classification, one individual per strain was randomly selected as test data and the classifier was trained on the remaining data. The test data was then used to predict the accuracy. Cross-validation was performed using all the members of the training data. The accuracy of the model is the average score of the cross-validated model. For health classification, a similar model was used with 25% test data and 75% training data. The area under the receiver operating characteristics (AUROC), a probability curve based on the degree of separability, was also used as a performance measurement for this trained classifier along with average cross-validated accuracy.

## Results

### Composition of the bacterial community is variable across CC strains

**Fig. 1:**
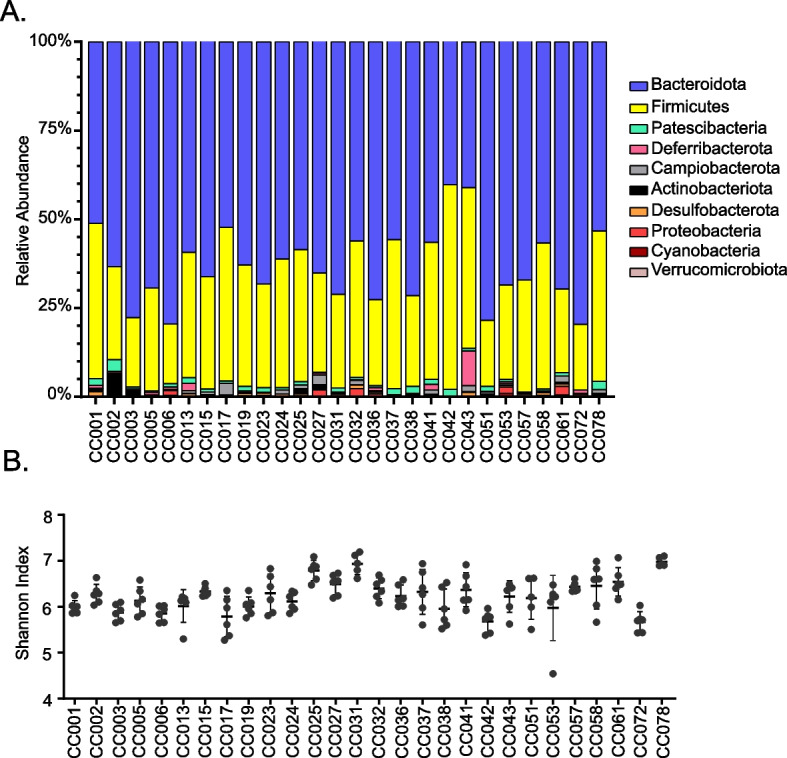
Diversity across the CC strain starts at the phylum Level (L2). **A** Bar graph of the relative bacterial abundance of all the phyla present in feces of 28 CC strains. Data represent 3 males and 3 females of each strain. **B** Within sample diversity measured by Shannon index. Bars represent mean and standard deviation for each strain. Kruskal–Wallis test was performed to analyze the statistical significance (*p* < 0.001)

One hundred sixty seven mice aged 8–12 weeks, 3 males and 3 females representing 28 CC strains (except for CC078—only two females), were included in this study. We noted a large variability in the bacterial composition of the feces across the CC strains that we studied (Fig. 1A). After assigning taxonomy using the SILVA 138 reference database, the Naïve Bayes classifier identified 10 phyla, 14 classes, 20 orders, 47 families, 113 genera, and 236 species with 99% sequence identity. At level 2, Bacteroidota (60.96%) was the most abundant phylum, followed by Firmicutes (34.52%) and Pastescibacteria (1.29%). The remaining seven phyla constituted less than 4% of the overall microbial composition and presence varied across strains (Table 1). *Muribaculaceae*, followed by *Lachnospiraceae* and *Alistipes*, were the top three genera by total counts. Only 20 genera were present in at least 95% of the samples and 37 genera were present in less than 25% of the samples (Table S1). This high variability both at the phylum and the genus level reflects the diversity of gut microbiome across the CC strains.

**Table 1:** Bacteroidota and Firmicutes were the most abundant phylum across the CC strains. Average, minimum, and maximum abundance of various phyla by strain, arranged in order through decreasing overall abundance

Phylum	Relative abundance by strain		
	Average	Minimum	Maximum
Bacteroidota	60.96%	40.21%	79.55%
Firmicutes	34.52%	16.80%	57.59%
Patescibacteria	1.29%	0.00%	3.19%
Deferribacterota	0.82%	0.00%	9.75%
Campilobacterota	0.70%	0.00%	3.27%
Actinobacteriota	0.66%	0.00%	6.05%
Desulfobacterota	0.52%	0.00%	1.38%
Proteobacteria	0.41%	0.00%	2.39%
Cyanobacteria	0.11%	0.00%	0.89%
Verrucomicrobiota	0.02%	0.00%	0.58%

### Bacterial communities within a strain are more similar than across strains

The Shannon index, a diversity measure based on richness, was calculated for individual animals (Fig. 1B). Individuals within a strain had similar diversity values while the values varied widely across the strains. The average diversity values for strains ranged from 5.7 to 7 with a mean value of 6.2 and a standard deviation of 0.3. The range of these values further demonstrates the diversity across the CC strains. Pairwise Kruskal–Wallis tests showed a significant difference (*P* < 0.001) in Shannon index across CC strains.

**Fig. 2:**
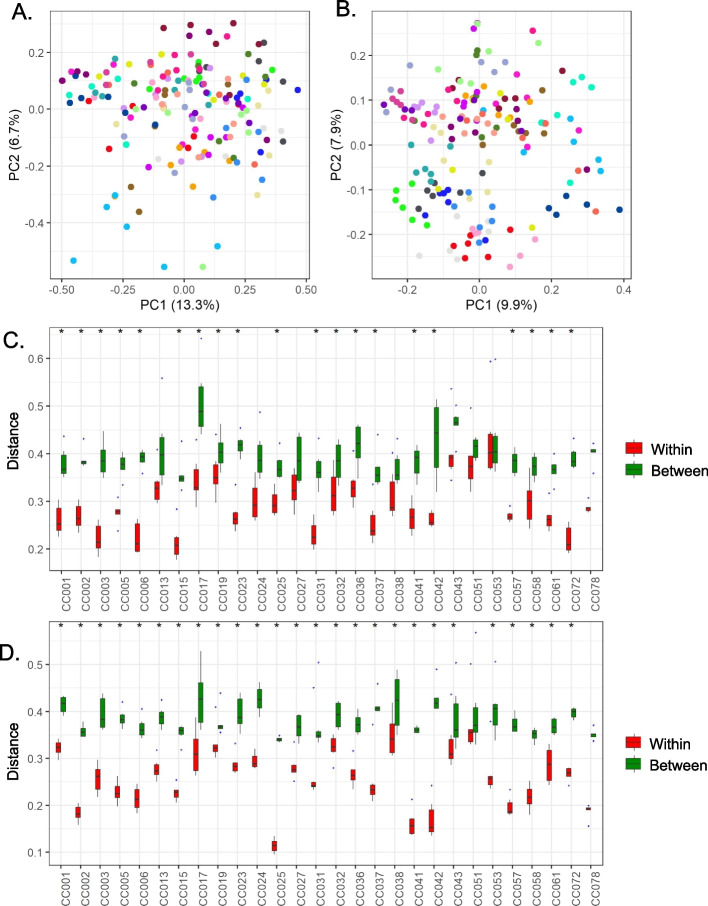
Analysis of beta diversity suggests fecal microbiota are more similar within CC strains than across CC strains. Principal component analysis (PCoA) 2D plots of between-sample dissimilarities measured by Bray–Curtis distance (**A**) and unweighted UniFrac distance (**B**). Each color represents a CC strain. Box plot of distances between fecal microbial communities obtained when comparing mice within and between strains of CC mice using Bray–Curtis distance (**C**) and Unweighted UniFrac distance (**D**). *P* < 0.05(*) for Wilcoxon signed rank test

A non-phylogenetic quantitative metric, Bray–Curtis dissimilarity, and phylogenetic qualitative metric, unweighted UniFrac, were used to measure beta diversity. Principal component analysis (PCOA) plots of Bray–Curtis dissimilarity and the unweighted UniFrac distance metric groups animals better within a strain than any other parameter in the metadata (Fig. 2A, B). To test for significance, pairwise PERMANOVA and ANOSIM analyses were performed. For Bray–Curtis, both analyses showed significant differences between the CC strains using the Kruskal–Wallis test. Unweighted UniFrac showed similar significance, but lower *R* and *F* values compared to Bray–Curtis (Table 2). This decrease could be explained by the complete absence of several ASV’s in some of the CC strains, as unweighted uniFrac takes presence/absence data into account.

**Table 2:** Bacterial composition differs across the CC strains. Distance between bacterial communities measured by Bray–Curtis and unweighted UniFrac metrics was used to analyze statistical significance between CC strains. Analysis of similarity (ANOSIM) and permutational multivariate analysis of variance (PERMANOVA) was performed with 999 permutations and the *R* and Pseudo-*F* test statistics are reported along with the *P*-values

Metric	Size	Groups	ANOSIM		PERMANOVA	
			*R*	*P*	*F*	*P*
Bray–Curtis	165	28	0.92	0.001	9.37	0.001
Unweighted UniFrac	165	28	0.48	0.001	5.08	0.001

We also noted increased spread within some of the CC strains in the PCOA plot. To determine the effect of individual variance within a CC strain, distances within a strain and across different strains were calculated and plotted (Fig. 2C, D). For both the metrics, within strain distances were significantly smaller compared with distances across the strains (*p* < 0.05 for Wilcoxon signed-rank test). This analysis showed that the individual variance within a strain had less impact on the significant differences observed in the microbial composition across different CC strains.

To determine the effect of littermates, we performed an analysis using Adonis. Initial litter cages had a small effect (6%) on microbiome composition, but this effect was negligible compared to the effect attributable to the CC strain itself (65%).

### Microbiome composition is a heritable trait

Broad-sense heritability was calculated for microbiome composition at the genus level. We assumed that the observed phenotype, microbial composition, is a result of unobserved genetic and environmental factors. The within-strain variance was used to calculate the environmental component and the variance across the CC strains used provided the total variance. The difference between the total and environmental variance produced the genetic component. Heritability was obtained by dividing the genetic variance by the total variance. For the genus-level data, the average heritability across the CC strain was 0.30 (Table S2). *Rikenellaceae*, *Acetatifactor*, and *Prevotellaceae* were the most heritable genera with scores more than 0.60 while *Turicibacter* and *Escherichia*-*Shigella* were the least heritable group. When sex was included as a variable, the average heritability increased to 0.39. Almost all the genera had a positive increase in scores showing that with a strain animals grouped better by sex. The strong heritability scores suggest the presence of genetic control in gut microbiome composition.

### QTL analysis reveals 17 significant associations

Differential abundance analysis was performed by analysis of composition of microbiomes (ANCOM). This analysis considers the relative abundances and structural zeros to greatly reduce false discovery rates. At the genus level, ANCOM identified 21 genera that were differentially abundant between the CC strains (Table S3). A heat map representing the relative abundance of genera identified by ANCOM analysis shows the diversity across CC strains (Fig. S2).

Genus-level abundance was considered a physical trait and QTL analysis was performed using R/qtl2. After grouping bacterial abundance by CC strain, any genera that were not present in at least four different strains were removed. Sex and kinship between the animals were considered covariates for the analysis. Peaks that crossed the 85% significant LOD score for individual genera were considered significant (Table S4). QTL analysis resulted in 17 statistically significant QTL peaks on 9 different chromosomes (Table 3). QTL peaks were named “Microbial Abundance in Feces” (Micabf) and numbered from 1 to 17. The heritability scores for these genera were all positive with an average of 0.31. The 1.8 peak drop confidence interval was calculated for each peak. The average length of QTL regions was 6.77 Mb, consistent with previous studies involving CC population [34, 62]. Genus *Peptococcus* and *Alloprevotella* had QTLs on two and three different chromosomes respectively. Genera *Muribaculaceae* (Micabf3) and *Erysipelotrichaceae* (Micabf9) were associated with regions adjacent to each other on chromosome 2. Similarly, genus *Ruminococcaceae* (Micabf14) and genus *Peptococcus* (Micabf16) were associated with regions adjacent to each other on chromosome 9.

**Table 3:**
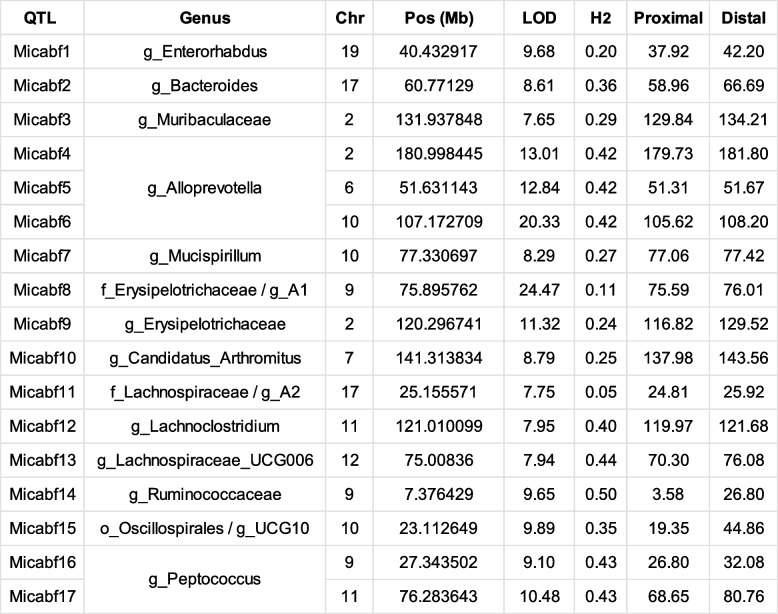
Analysis of bacterial abundance identifies numerous QTL. Bacterial abundance in the feces of 28 CC strains was used to identify QTL using rQTL2. 17 statistically significant peaks were identified on 9 different chromosomes. *Chr*, chromosome; *Pos*, position; *LOD*, logarithm of the odds; *H2*, broad sense heritability; proximal and distal regions are in Mbp

### Genes within the QTL regions are associated with important functions

**Fig. 3:**
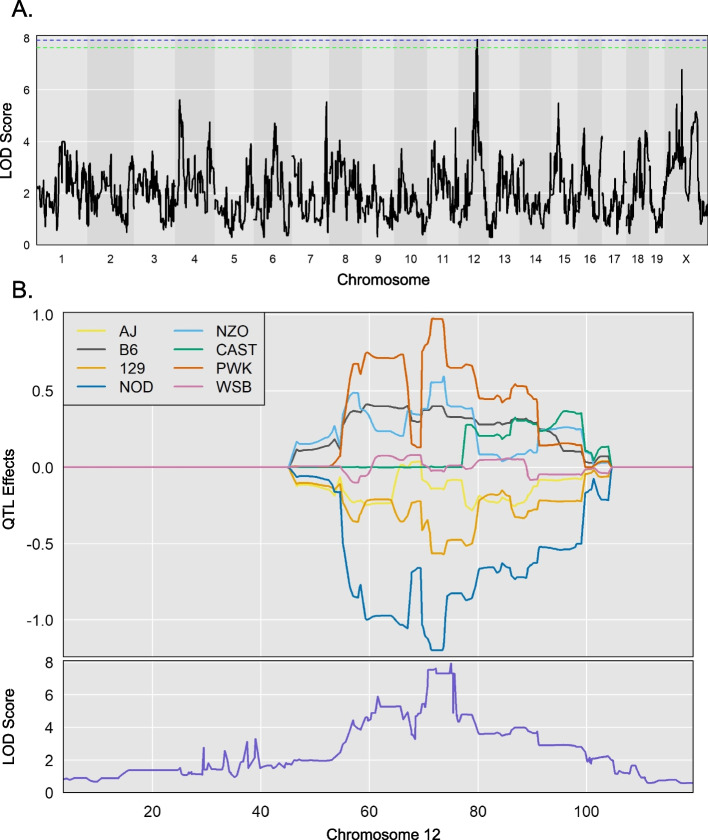
Genus *Lachnospiraceae* has a significant peak on chromosome 12. **A** LOD plot for rank transformed relative abundance values for genus *Lachnospiraceae.* The dotted green line represents 85% significance and blue line represents 90% significance. **B** Founder effect plot for genus *Lachnospiraceae* on chromosome 12

We wanted to utilize the millions of SNPs present in the CC genome to further shortlist and correlate the genes to bacterial abundance in the microbiome of different mouse strains. The Mouse Genome Informatics (MGI) database was used to collect all the protein-coding genes and other features from each QTL interval (Table S5). We picked genus *Lachnospiraceae* UCG-006, which was differentially abundant between the healthy and sick groups after infection, for further study. The QTL plot for this genus displayed a significant peak on chromosome 12 (Fig. 3A). This 6 Mb region contains 45 protein-coding genes. The founder effect plot (Fig. 3B) suggested that CC strains with NOD and 129S1 allele in this region had lower abundance of genus *Lachnospiraceae* UCG-006 compared to other founder strains. These two alleles could act independently, or the same variant could be causing this effect in different CC strains.

We shortlisted all variants that matched this founder allele pattern and identified 1490 variants representing 31 of the 45 protein-coding genes. Then, we used the variant effect predictor to identify variants that had high impact on the protein structure. This analysis identified three genes with missense mutations and one gene with a 3′ UTR variant (Table 4). Which of these high-impact mutations is involved in determining the composition of genus *Lachnospiraceae* in the CC strains requires further experimentation. We repeated this process for all the other significant peaks and found high-impact variants for 11 regions (Table S6).

**Table 4:**
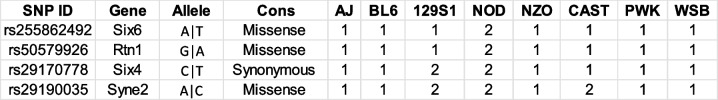
Top SNP’s for genus *Lachnospiraceae* on chromosome 12. Founder allele pattern was used to filter snp’s within the significant interval in chromosome 12. Variant effect predictor was used to calculate the consequence for the variants

In order to identify pathways associated with the variants we identified, we used the Genecards database to identify putative functions for high-impact variants. Several of these genes, including Ralbp1 [63], Pfas [64], Tyro3 [65], Opcml [66, 67], Syne2 [68], Six4 [69], Tll2 [70], and Slc23a2 [65], were previously implicated in gut microbiome abundance, metabolic traits, and several intestinal diseases. A comprehensive list of all the gene summaries, previous GWAS analysis, and pathways can be found in Table S7.

We further analyzed these genes for enrichment using enrichr. This database has more than 400,000 terms from 200 different libraries. Any term with a *p* value of < 0.05 was considered significant. These genes were enriched for terms associated with BMI, metabolic traits, immune response, biochemical pathways, and neurological conditions (Table S8). Association of these variants with significant biological processes suggests the importance of these genes in determining the composition of the gut microbiome.

### Differential abundance of genera Parasutterella and Lachnospiraceae-UCG-006 correlated with STm infection outcome

**Fig. 4:**
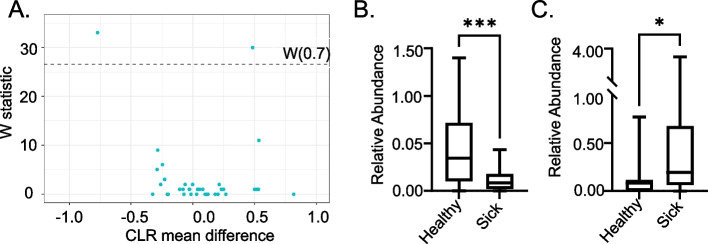
ANCOM identifies genera that are differentially abundant in healthy versus sick strains. Genera (L6) differentially abundant between healthy and sick groups were identified by ANCOM. W-Statistic score above 0.7 was considered significant (**A**). Genus *Lachnospiraceae UCG-006*, class Clostridia, phylum Firmicutes, is present in higher abundance in CC strains that remained healthy after STm infection (**B**). Genus *Parasutterella*, class Betaproteobacteria, phylum Proteobacteria, were in lower abundance in animals that remained healthy after STm infection (**C**). *P* < 0.05 (*) and *P* < 0.001 (***) for Student’s *t*-test

A subset of our CC strains were infected with *Salmonella enterica* serotype Typhimurium (STm) 2 days after the collection of feces for this study. We wanted to determine if the abundance of different genera influenced the infection outcome. Forty-eight animals from 8 different CC strains, 3 males and 3 females per strain, were included in this analysis. Four strains with a median survival time of less than 7 days were classified as “sick” and the other four strains that survived to day 7 post-STm infection without clinical signs were classified as “healthy.” ANCOM analysis on pre-infection feces between these two groups was performed after reducing the counts to the genus level. This analysis identified two genera as differentially abundant in the pre-infection microbiomes of healthy and sick mice (Fig. 4A and Table S3). Increased abundance of genus *Lachnospiraceae UCG-006* and decreased abundance of genus *Parasutterella* were correlated with healthy outcome after infection with STm (Fig. 4B, C).

### Machine learning accurately predicts the metadata groups

We used bacterial composition to predict the metadata groups (CC strain and healthy vs. sick after STm infection) using a machine learning classifier. The bacterial composition data at the genus level was randomly split into training and test sets, and the training set was used to train a random forest classifier. Then, each test sample was used to predict the metadata group based on its fecal microbial composition. Cross-validations were performed during parameter optimization and feature selection to tune the model.

**Fig. 5:**
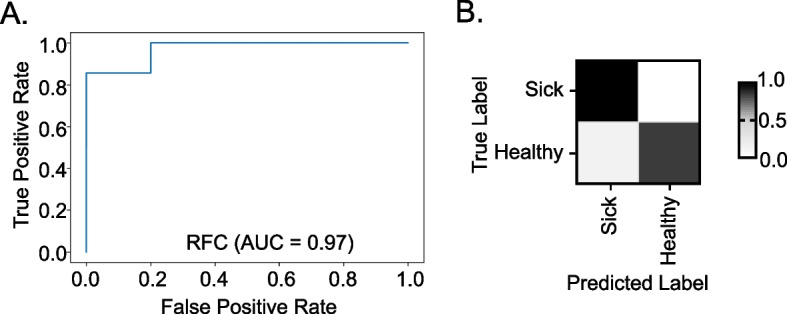
Machine learning algorithm predicts infection outcome. The area under the random operation curve (AUROC) plot for the trained random forest health classifier. AUC (area under the curve) = 0.97 (**A**). Model accuracy plot for the classifier (**B**)

First, for the CC strain data, the classifier assigned blinded test data to the right CC strain group with 90% accuracy and a cross-validation average accuracy of 0.89. The mislabeled CC strain groups were mostly random (Fig S3). This mislabeling may be due to the small sample size used (*n* = 5) when training the classifier. The top features identified from the model were genera *Parabacteroides*, *Rikenellaceae*, and *Odoribacter* (Table S9). The importance score of the top 5 features from the model added up to only 0.13 again illustrating diversity in the microbiome composition across the CC strains. Second, for the STm infection outcome data, the classifier mislabeled some of the healthy animals into the sick group leading to an overall accuracy of 90% and an AUROC of 0.97 for the model (Fig. 5A, B). Genera *Lachnospiraceae UCG-006 and Parasutterella,* which were also identified by ANCOM, *along* with genus *Preveotellaceae_UCG-001*, were the top features for this classifier. The importance of the top 5 features added up to 0.26. This higher value indicates that infection outcome prediction is more tightly controlled by fewer genera than the strain classifier (Table S9). The higher accuracy rate of these classifiers shows that machine learning can be successfully used in predicting both strain and infection outcome using the microbiome data.

## Discussion

Growing evidence suggests a complex interaction between host genes and the microbiome [26, 29, 71, 72]. Furthermore, the combined effects of host genetics and microbiome composition on several phenotypes including sleep, anxiety, and liver damage have previously been identified [34, 36, 37]. The effect of diet, environment, early exposure to certain microbes, and the use of antibiotics makes it challenging to study these interactions in humans.

We analyzed the bacterial composition of the feces collected from 28 genetically diverse strains of CC mice. Despite identical housing, food, and husbandry, fecal microbial communities differed significantly across CC strains, beginning at the phylum level. The most abundant phylum, Bacteroidetes, had a relative abundance that varied from 40 to 80% across CC strains. The second most abundant phylum, the Firmicutes, had a relative abundance that varied between 17 and 58% across CC strains (Table 1). But Firmicutes were the most abundant phylum in the previously published CC cecal microbiome data [34, 73]. This difference is likely due to sample collection site differences (feces vs. cecum) [34, 73]. Comparing all the datasets in the Murine Microbiome Database, Firmicutes were the most abundant phylum in the cecum (70%), while Bacteroidetes were the most abundant phylum in the feces (46%) [74]. In our study, the other eight observed phyla were absent in as few as one strain and as many as 26 of the 28 strains. This difference across the CC strains was also noticeable in the alpha diversity metric, Shannon’s index (Fig. 1B). Diversity starting at such a high level is interesting and warrants consideration of the microbiome as an important factor when comparing phenotypic outcomes from different inbred strains.

Animals within a CC strain tended to have more similar bacterial composition profiles in the feces than animals from different CC strains. The PCOA plots for two of the diversity metrics (Bray–Curtis and unweighted UniFrac) grouped mice better by strain than by any other observed parameter. PERMANOVA and ANSOIM analysis further confirmed significant differences across CC strains. Next, we tested whether these differences were caused by the variations in individual animals within a strain. We calculated the distance in individuals within a strain and between strains. For both diversity metrics, the between-strain distances were significantly higher than the individual distance within the strains. Similar observation was made in the microbiome study involving CC founders [73]. This close association of bacteria within a strain and diversity across the strain makes CC mice a great tool for studying the effect of host genetics on the gut microbiome.

Keeping in mind the limitations of 16S rRNA gene sequencing in prediction of species-level data, we identified genetic associations with the abundance of certain microbes at the genus level. All genera had positive heritability scores with more than half of the genera having a score of 0.25 and above. These broad sense heritability values do not take the founder genotype into account. When sex was included as a variable, heritability scores increased suggesting tighter control by sex within CC strains. Given the influence of environment and diet on gut microbiome, these values suggest that microbiome composition has a considerable heritability [75].

We also employed a machine-learning algorithm to predict the effect of host genotype on the microbiome. The Random Forest classifier had an overall accuracy of 90% and cross-validation score of 0.89 in matching microbiome composition to the correct CC strain. The accuracy of the classifier to properly predict the CC strain based on the genus-level bacterial composition data provides further evidence for the influence of host genetics on microbial abundance.

For successful genetic association and QTL mapping, a phenotype must be diverse across the CC strains. Using R/qtl2 analysis, we identified 17 significant peaks on 9 different chromosomes linked to 14 different genera. The 1.8 peak drop confidence interval ranged from 0.3 to 26 Mb with a median range of 4.36 Mb. These short QTL regions, given the size of the population included in this study, demonstrate the power of QTL mapping with CC strains.

Several similar studies in mice have identified QTL linked to the presence of particular members of the microbiota. Micabf6, a region on chromosome 10 that we identify as linked to the presence of genus *Alloprevotella*, overlapped with six QTL regions identified in the Advanced Intercross population as linked to particular members of the microbiota. These QTL regions were associated with the presence of *Coriobacteriales*, Streptococcaceae, and *Lactococcus* [29]. Micaf8 on chromosome 9 also overlaps with a QTL identified from the Advanced intercross population for genus *Barnesiella*. Micabf11 on chromosome 17 associated with the presence of *Lachnospiraceae* in our study, matches three different QTL regions identified in the BXD population study [31]. These three regions were associated with the presence of Bacillales and Staphylococcus.

The only other QTL study involving CC strains [34], examined microbial abundance in the cecum. Three QTL associated with microbial matched QTL identified in this study. Genetic regions associated with genera *Caminicella*, *Turicibacter*, and *Tannerella* overlapped with Micabf8 (Chr 9, Erysipelotrichaceae), Micabf7 (Chr 10, Mucispirillum), and Micabf1 (Chr 19, Enterorhabdus), respectively. Even though the genera associated with each of these overlapping regions did not match, these regions may be influencing microbial abundance by a common mechanism.

For individual QTL identified in this study, we looked closely into the genes with structural variants in each interval. Many genes in the QTL we identified had critical roles in important body functions including metabolism and immunity (Table S7). KEG pathway enrichment analysis for these genes picked several important metabolic pathways including protein absorption, sucrose metabolism, and inositol phosphate metabolism. Similarly, mammalian phenotype enrichment identified genes that are involved in colon morphology, as well as several immune and enzyme modulators. We further shortlisted top candidate genes based on a previous association with human disease (Table 5). Most of these genes were previously associated with gut microbiota composition. These intervals also contained genes associated with body mass index (BMI), intestinal bowel disease (IBD), and ulcerative colitis (UC), suggesting a function for these genes in the intestinal tract [58].

**Table 5:**
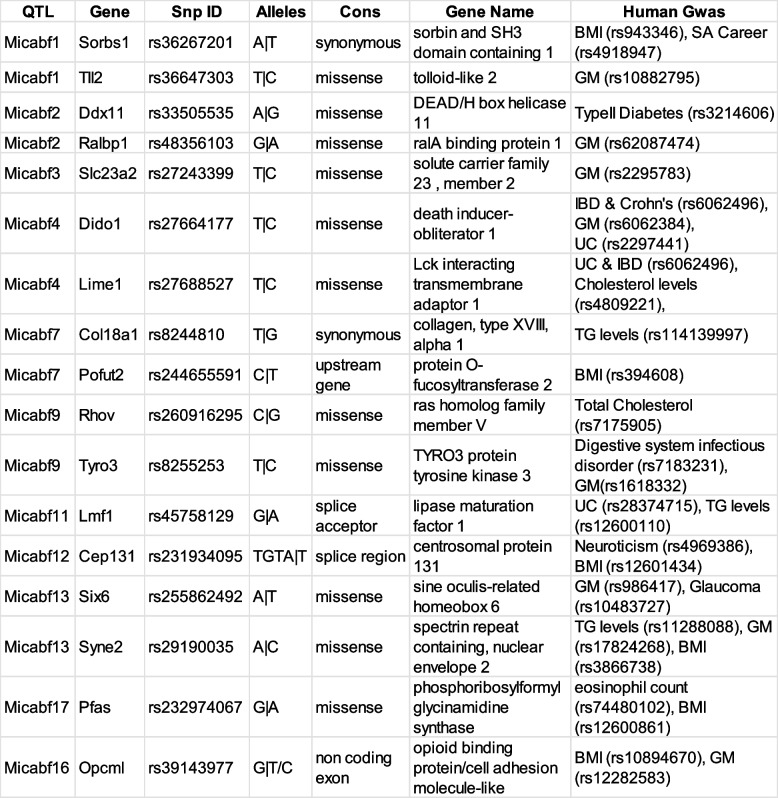
Top Genes of Interest for all QTL regions. Shortlisted protein-coding genes based on founder allele pattern, consequence, and previous GWAS hits

Given their location in the intestine and their ability to produce and modify several metabolites, various bacterial species have been proposed or used as probiotics for conditions including obesity, diarrhea, diabetes, *Clostridium difficile* infection, IBD, and neurological diseases [76–81]. Many of these probiotics have failed to produce the desired results at the population level [82–86]. The complexity of and interplay between host genetics and microbial composition may explain why simple supplementation with a given bacterial species has not been particularly successful in genetically diverse populations such as humans.

A decrease in beneficial microbes, enrichment of pathogens, and imbalances of metabolites produced by microbes can alter the outcome of diseases [87]. We wanted to identify correlations between pre-infection bacterial composition and the outcome of STm infection. Previous murine studies have successfully employed machine-learning algorithms to predict the outcome of phenotypes including memory, anxiety, and AOM-induced toxicity [35–37]. We employed a similar random forest classifier to predict the CC genotype with respect to microbial composition. The algorithm we used also identified several genera that were important in predicting the outcome of infection with STm. Genus *Parasutterella* was the top feature that allowed the algorithm to differentiate between animals that remained healthy versus those that became ill after STm infection. To further shortlist genera that were differentially abundant between the sick and healthy groups, we employed ANCOM. This analysis identified *Lachnospiraceae UCG-006* in addition to *Parasutterella* as significantly different between the two groups.

*Parasutterella* is also found in other host species including humans, rats, dogs, pigs, chicken, turkeys, and calves [88]. Changes in the relative abundance of this genus have been reported in several diseases. *Parasutterella* are increased in submucosal tissues of patients with advanced Crohn’s disease [89] and are associated with pancreatitis in rats [90]. Increased abundance of *Parasutterella* is also associated with depression and major depressive disorder [91, 92]. Furthermore, increased abundance of *Parasutterella* was linked to the genesis and development of irritable bowel syndrome (IBS) and is associated with chronic intestinal inflammation in patients with IBS [93].

*Parasutterella* produce succinate as a fermentative end product and also alter the production of several microbial-derived metabolites involved in bile acid maintenance, tyrosine, tryptophan, and cholesterol metabolism [88]. In our experiments, genus *Parasutterella* was abundant in the pre-infection fecal microbiota of CC strains that developed clinical signs after infection with STm. Opportunistic pathogens including, Enterohemorrhagic *Escherichia coli* and *Clostridium difficile*, sense succinate produced by the commensal gut microbiota through a transcriptional regulator, catabolite repressor/activator (*cra*) [94, 95]. STm also utilizes succinate produced by intestinal commensals [96] and *cra* gene activation is important for STm pathogenesis [97]. Sensing of succinate results in the activation of STm virulence genes, including activating the *Salmonella* pathogenicity island 2 type III secretion system (SPI2 T3SS), leading to increased pathogenicity [98, 99]. Thus, succinate produced by *Parasutterella* may be sensed by STm leading to increased virulence and development of severe clinical symptoms in CC strains that harbor this organism in the intestinal tract. This hypothesis remains to be tested.

The family Lachnospiraceae is a core member of the gut microbiota in both mice and humans [74, 100]. Despite being one of the main producers of short-chain fatty acids (SCFA) in the intestine and helping with metabolism [101], the role of family Lachnospiraceae is controversial. The relative abundance of multiple genera in this family can both positively and negatively influence several diseases, including obesity, diabetes, IBD, and depressive syndrome [102]. In our study, the relative abundance of genus *Lachnospiraceae UCG-006* is associated with a positive health outcome after STm infection. Increased relative abundance of genus *Lachnospiraceae UCG-006* is correlated with positive outcomes in several diseases including colon cancer, IBD, LPS-induced inflammation, and colitis ([103–106]. Butyrate, a SCFA, can be utilized by intestinal epithelial cells as an energy source [107, 108]. Butyrate also reduces the colonization and virulence of several *Salmonella* sp*.* including Typhimurium [109–113]. It is possible that SCFA produced by *Lachnospiraceae* species may reduce the initial intestinal colonization of STm, leading to a positive outcome after infection.

## Conclusions

Diversity and homeostasis are key to the proper functioning of our gut microbiome. Changes in this delicate balance can lead to metabolic abnormalities, intestinal inflammation, infection by pathogens, autoimmunity, and neurological diseases. By controlling the diet, environment, and varying host genetics, we identified multiple regions of the mouse genome that are associated with intestinal colonization by specific microbes. Despite the small sample size, we successfully used machine learning tools to predict the metadata columns. With publicly available bacterial composition data and advancements in the artificial intelligence field, more machine learning tools can be employed to develop microbes as biomarkers for disease prediction. Future genetic modification and gnotobiotic studies in murine models such as the CC panel will be useful to convert the correlated genes to causal candidates for therapeutic application. A clear understanding of the role of environment, diet, and host genetics will provide a basis for using microbes as a personalized therapeutic tool to prevent, diagnose and treat various body conditions and diseases.

## Supplementary Information

**Additional file 1:** **Figure S1.** Sequence counts significantly decreased after DADA2 processing. (A) Raw sequence counts from Illumina paired end sequencing. (B) Sequence counts after filtering, denoising, chimeric removal and merging using DADA2 pipeline.

**Additional file 2:** **Figure S2.** Diverse genus communities are observed across the CC strains. The heatmap of log transformed relative abundances of top differentially abundant genera between CC strains as identified by ANCOM.

**Additional file 3:** **Figure S3.** Machine learning algorithm accurately predicts the metadata columns. Random forest classifier was trained using the bacterial composition data and predict the CC strain type.

**Additional file 4:** **Figure S4.** Plots for all the significant QTLs. A. LOD plot for rank transformed relative abundance values for significant genera. The dotted lines (Green – 85%, Blue – 90%, Red – 95%) represent significant thresholds calculated by 999 permutations. B. Founder effect plot and LOD plot for the significant chromosome. C. Genotype X Phenotype plot for the highest SNP on the significant chromosome.

**Additional file 5:** **Table S1.** Genera counts and Frequency.

**Additional file 6:** **Table S2.** Broad sense heritability scores.

**Additional file 7:** **Table S3.** ANCOM analysis.

**Additional file 8:** **Table S4.** LOD scores.

**Additional file 9:** **Table S5.** All protein coding genes and mouse QTL's within each significant region.

**Additional file 10:** **Table S6.** Top Snps in each QTL.

**Additional file 11:** **Table S7.** Important Genes, GWAS associations and pathways.

**Additional file 12:** **Table S8.** Enrichment Analysis.

**Additional file 13:** **Table S9.** Machine Learning Importance scores.

**Additional file 14:** **Table S10.** ASVs.

## Data Availability

All the raw data files and supplemental information is publicly available at https://figshare.com/articles/dataset/Raw_FastQ_files/21964754 and https://figshare.com/articles/journal_contribution/Supplemental_Information/21964745. ASVs obtained for individual mice along with the metadata are available in Table S10. QTL, Founder effect, and Genotype vs phenotype plots for all the significant QTLs are available as supplementary file (Fig S4).
