# Supplementary figures and images for: Using the collaborative cross to identify the role of host genetics in defining the murine gut microbiome
Source: Microbiome. 2023 Jul 8;11:149. doi: 10.1186/s40168-023-01552-8 (PMC10329326; doi:10.1186/s40168-023-01552-8)

## Slide 1
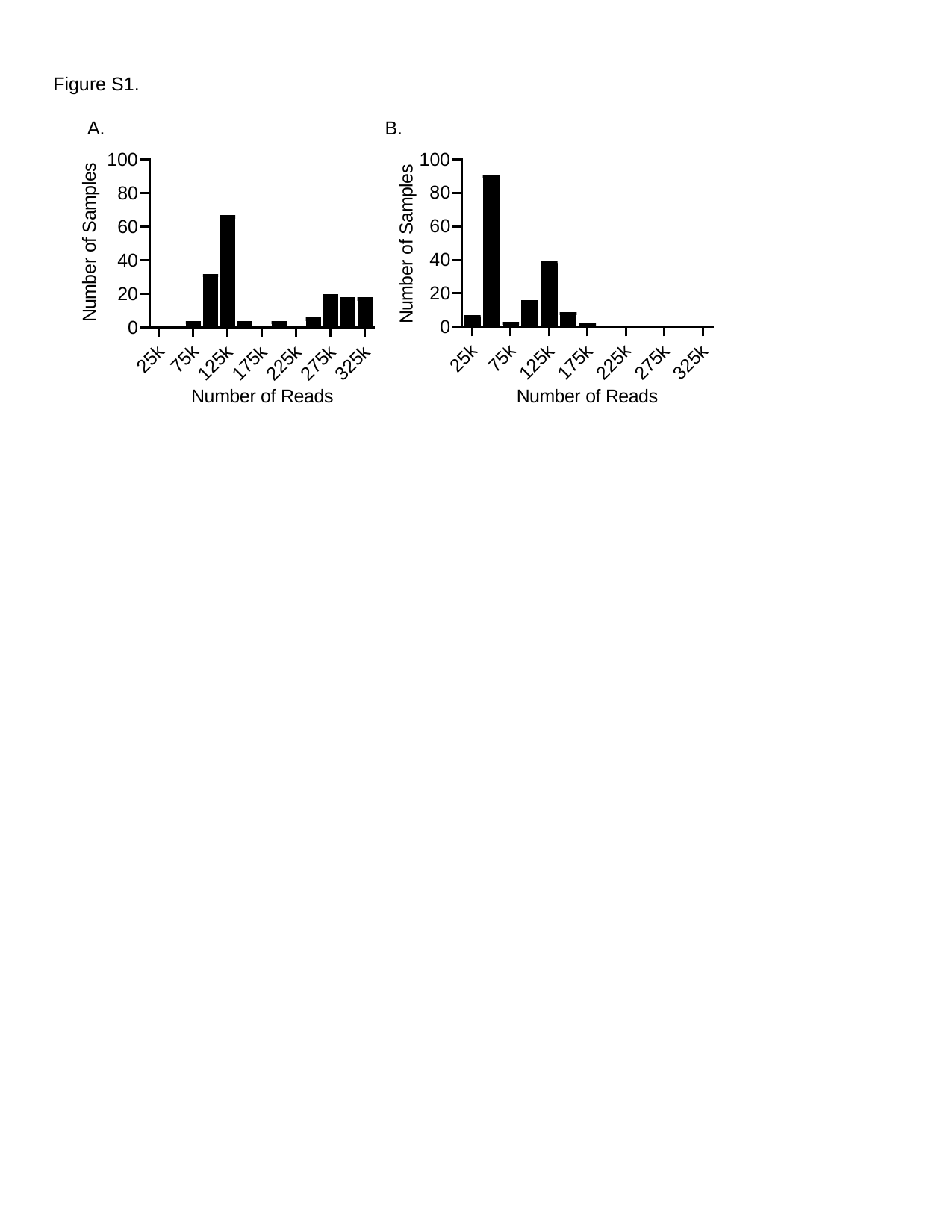

Figure S1.
A.
B.

Supplement: Supplementary file 2 — Additional file 1: Figure S1. Sequence counts significantly decreased after DADA2 processing. (A) Raw sequence counts from Illumina paired end sequencing. (B) Sequence counts after filtering, denoising, chimeric removal and merging using DADA2 pipeline. [file 40168_2023_1552_MOESM1_ESM.pptx]

## Slide 1
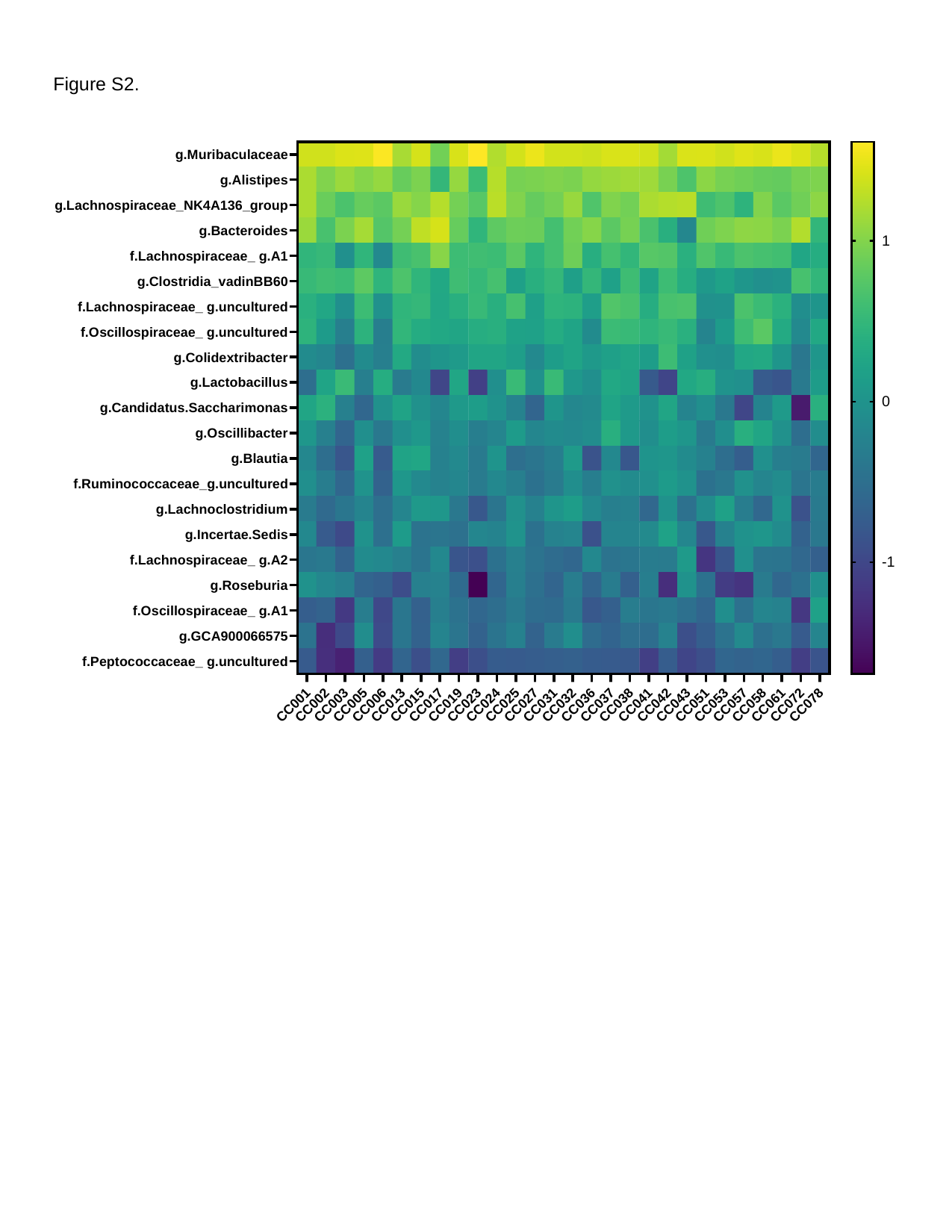

Figure S2.

Supplement: Supplementary file 3 — Additional file 2: Figure S2. Diverse genus communities are observed across the CC strains. The heatmap of log transformed relative abundances of top differentially abundant genera between CC strains as identified by ANCOM. [file 40168_2023_1552_MOESM2_ESM.pptx]

## Slide 1
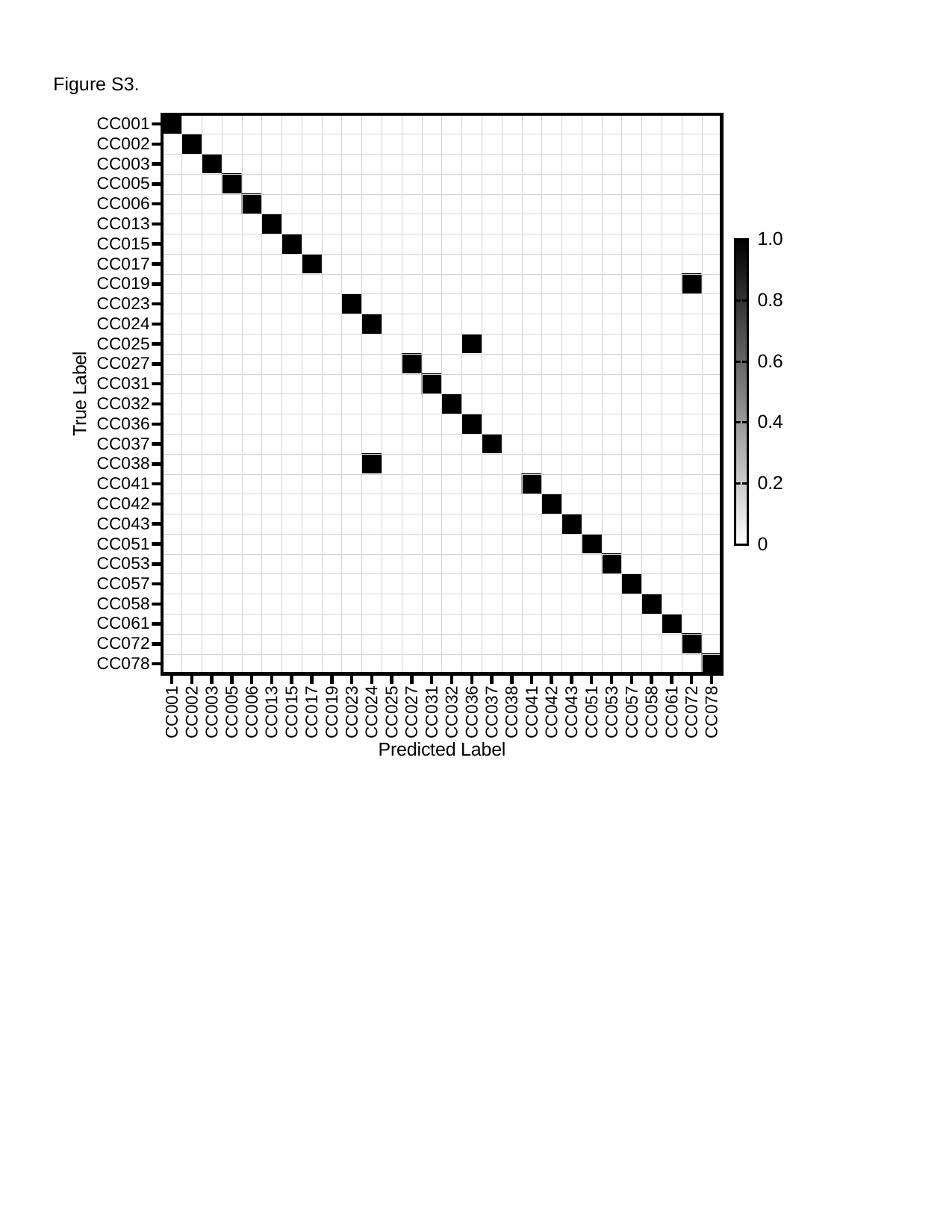

Figure S3.

Supplement: Supplementary file 4 — Additional file 3: Figure S3. Machine learning algorithm accurately predicts the metadata columns. Random forest classifier was trained using the bacterial composition data and predict the CC strain type. [file 40168_2023_1552_MOESM3_ESM.pptx]
