# Supplementary material for: Using the collaborative cross to identify the role of host genetics in defining the murine gut microbiome
Source: Microbiome. 2023 Jul 8;11:149. doi: 10.1186/s40168-023-01552-8 (PMC10329326; doi:10.1186/s40168-023-01552-8)

Supplementary file: A. LOD plot for rank transformed relative abundance values for significant genera. The dotted lines (Green – 85%, Blue – 90%, Red – 95%) represent significant thresholds. B. Founder effect plot for the significant chromosomes. C. Genotype X Phenotype plot for the highest SNP on the significant chromosome.

Fig Q1: Micabf1 - g_Enterorhabdus

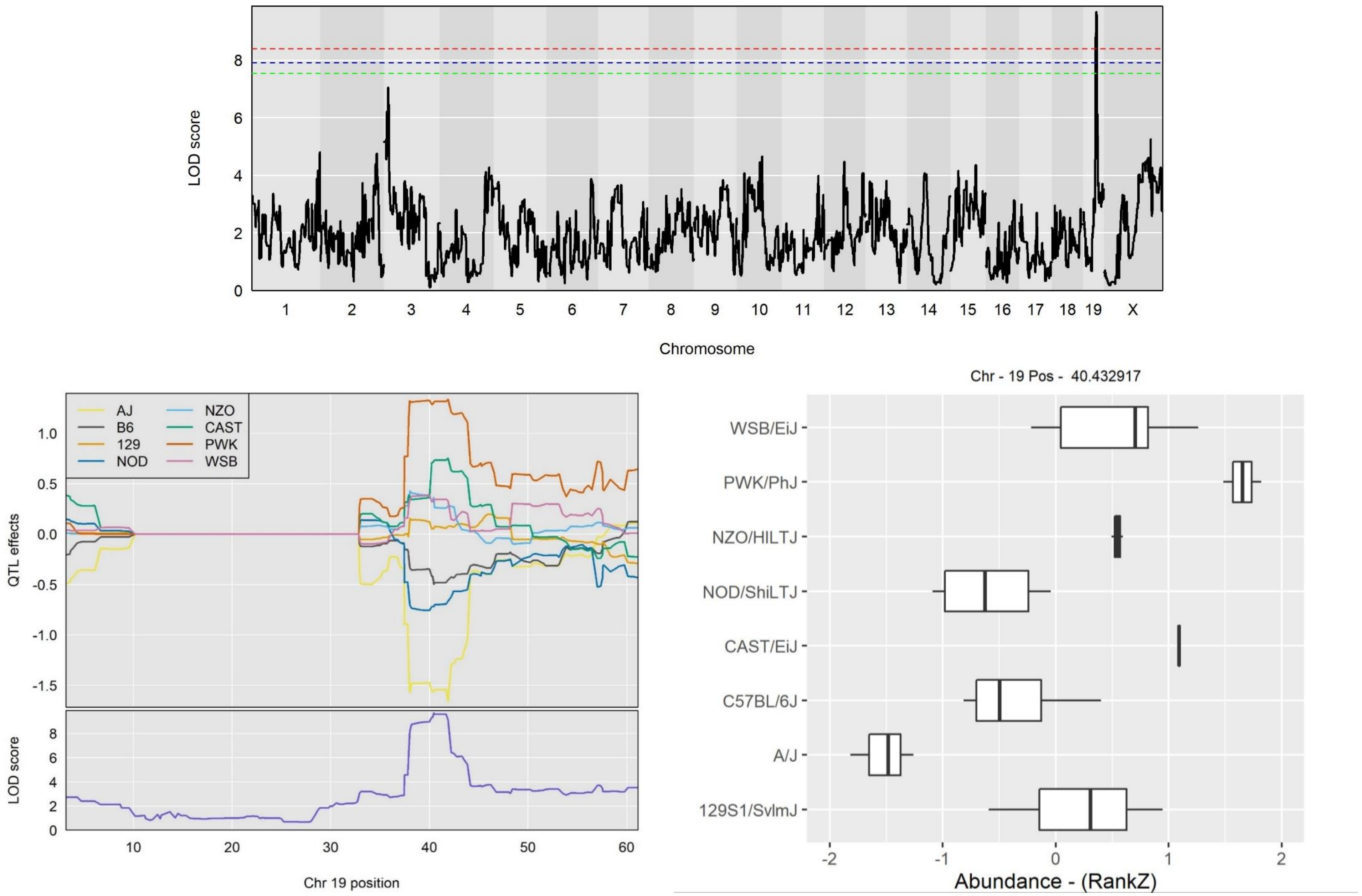

Fig Q2: Micabf2 - g_Bacteroides

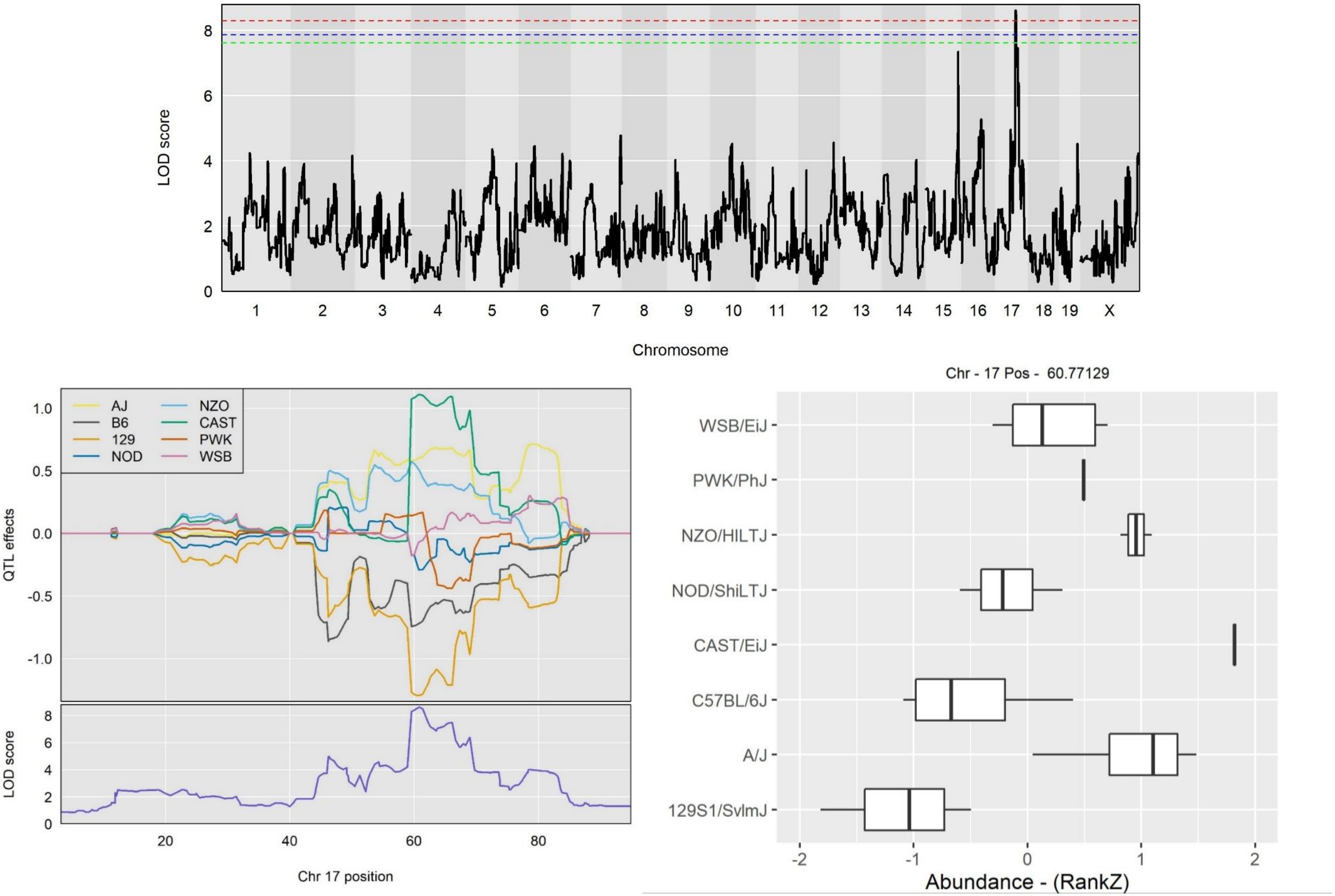

Fig Q3: Micabf3 - g_Muribaculaceae

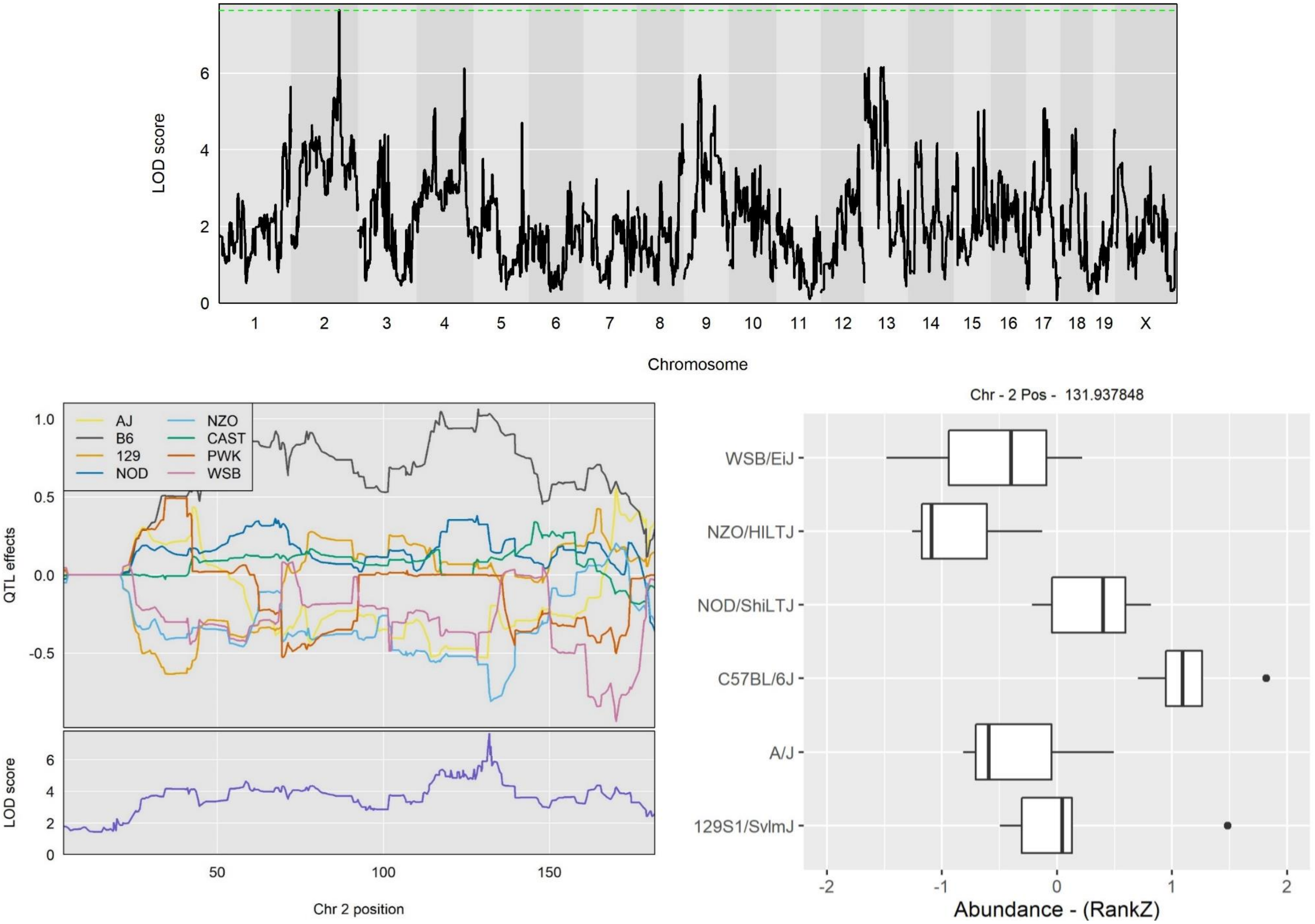

Fig Q4: Micabf4 - g_Alloprevotella

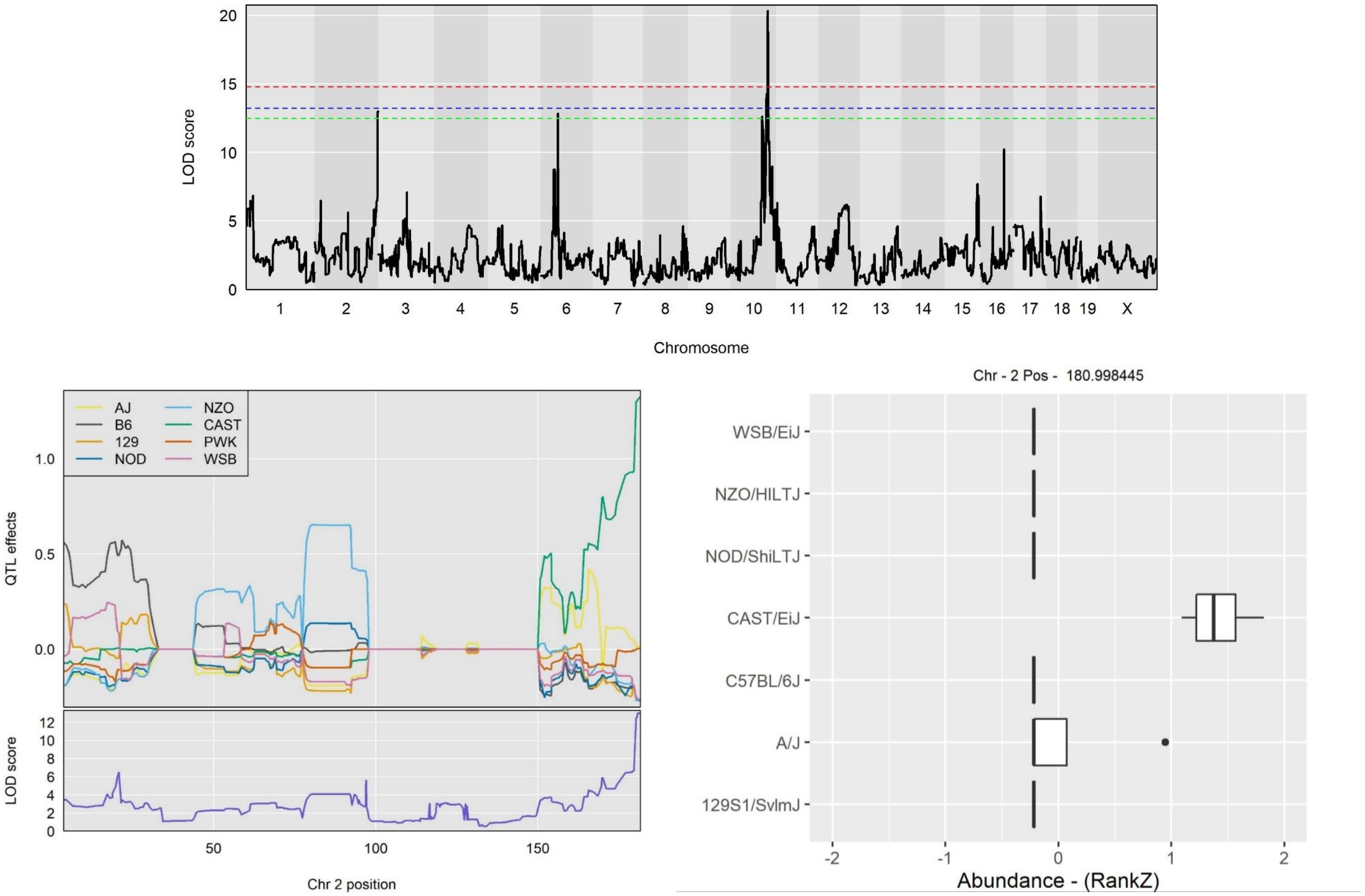

Fig Q5: Micabf5 - g_Alloprevotella

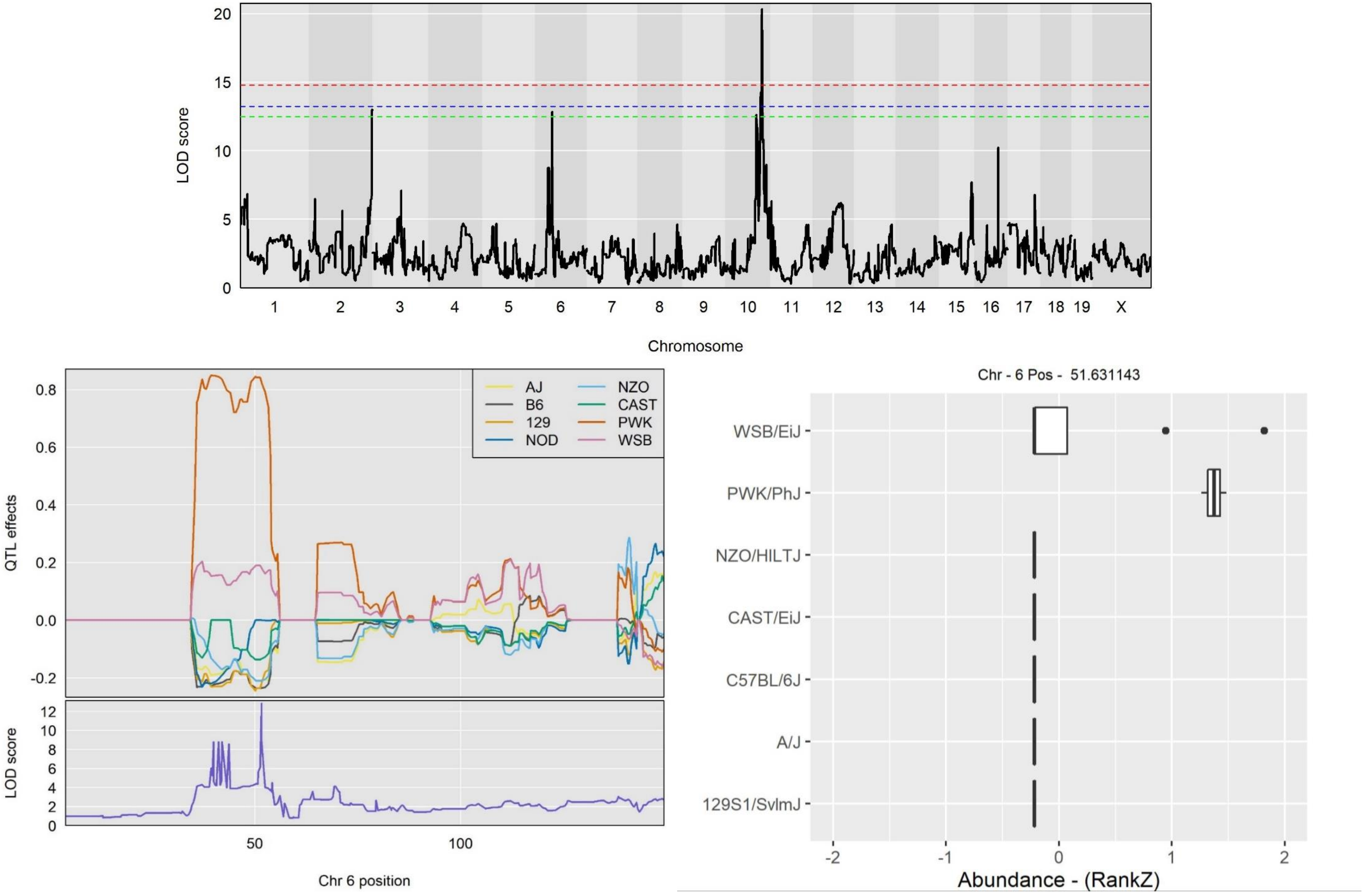

Fig Q6: Micabf6 - g_Alloprevotella

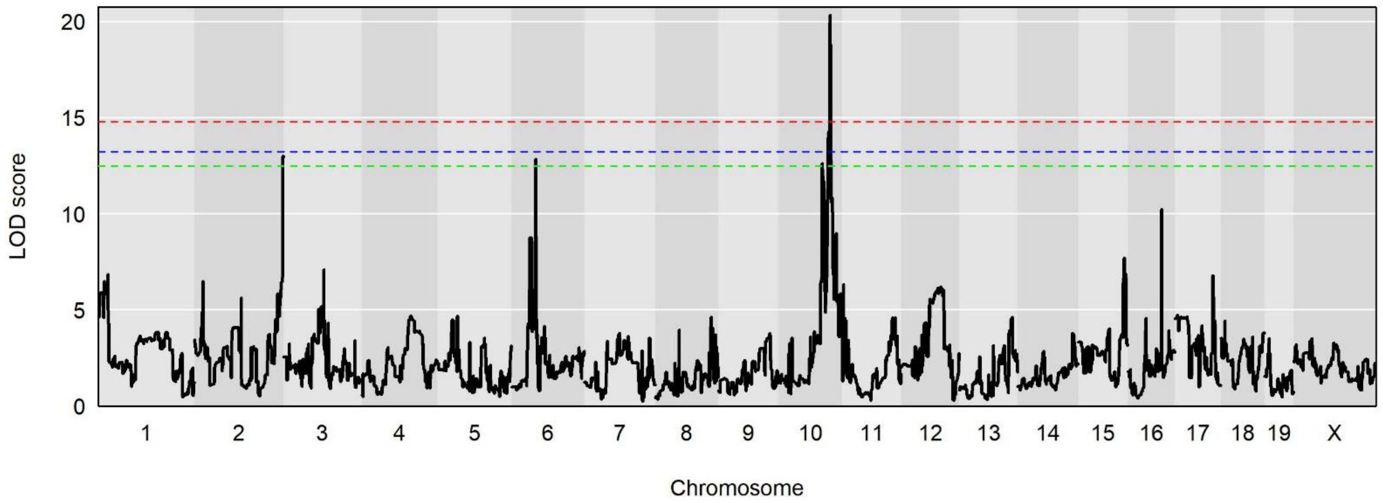

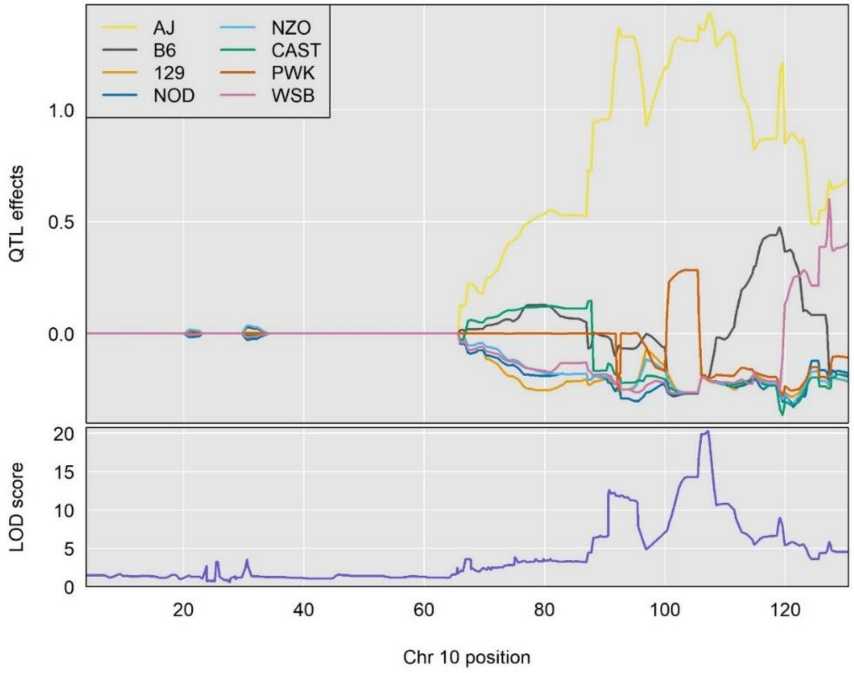

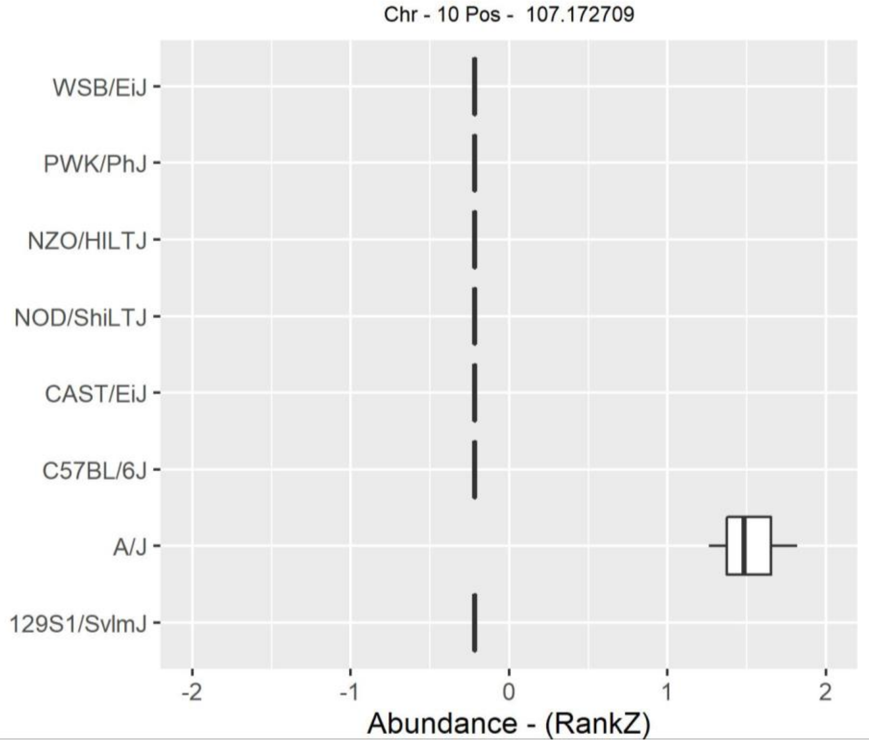

Fig Q7: Micabf7 - g_Mucispirillum

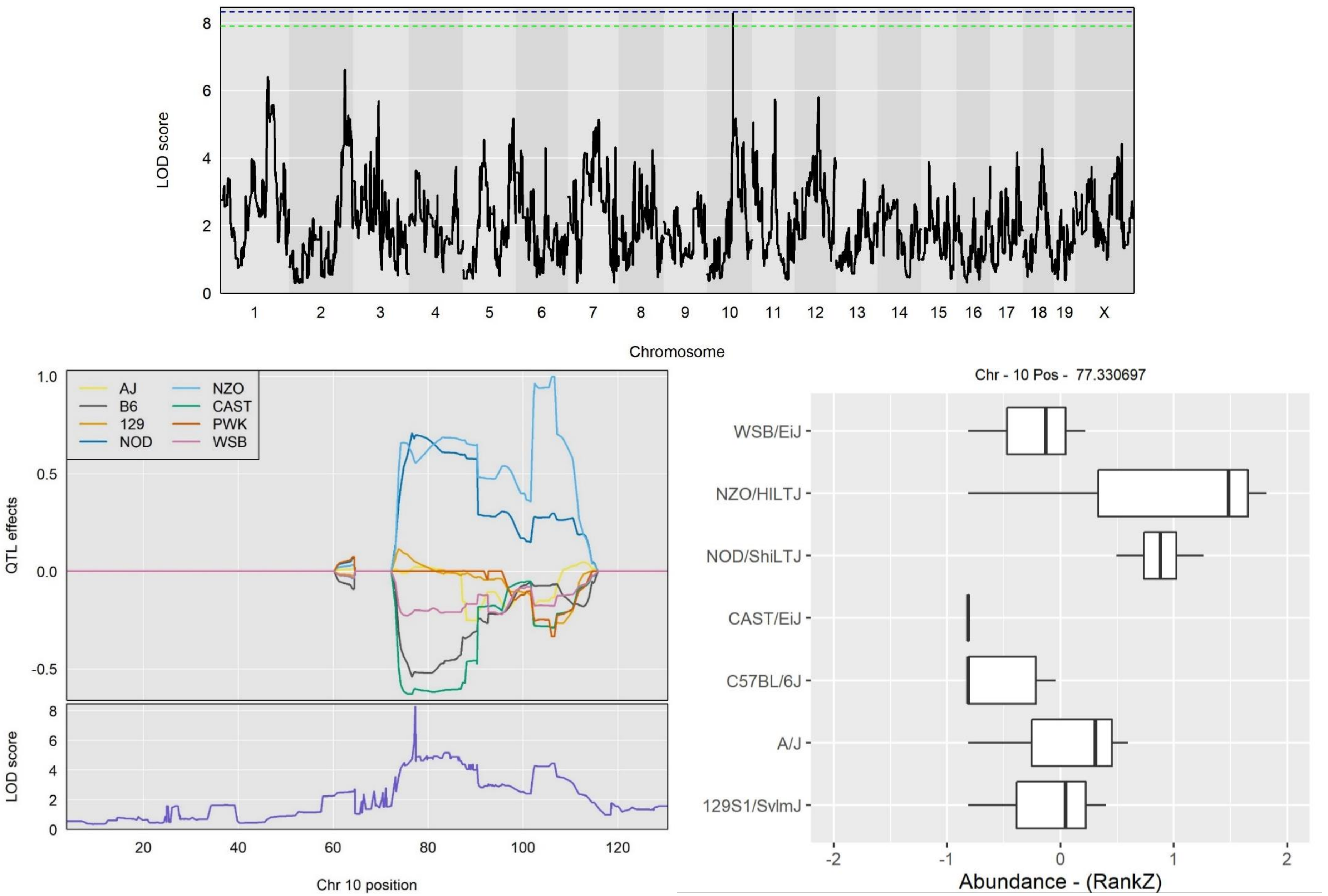

Fig Q8: Micabf8 - f_Erysipelotrichaceae / g_A1

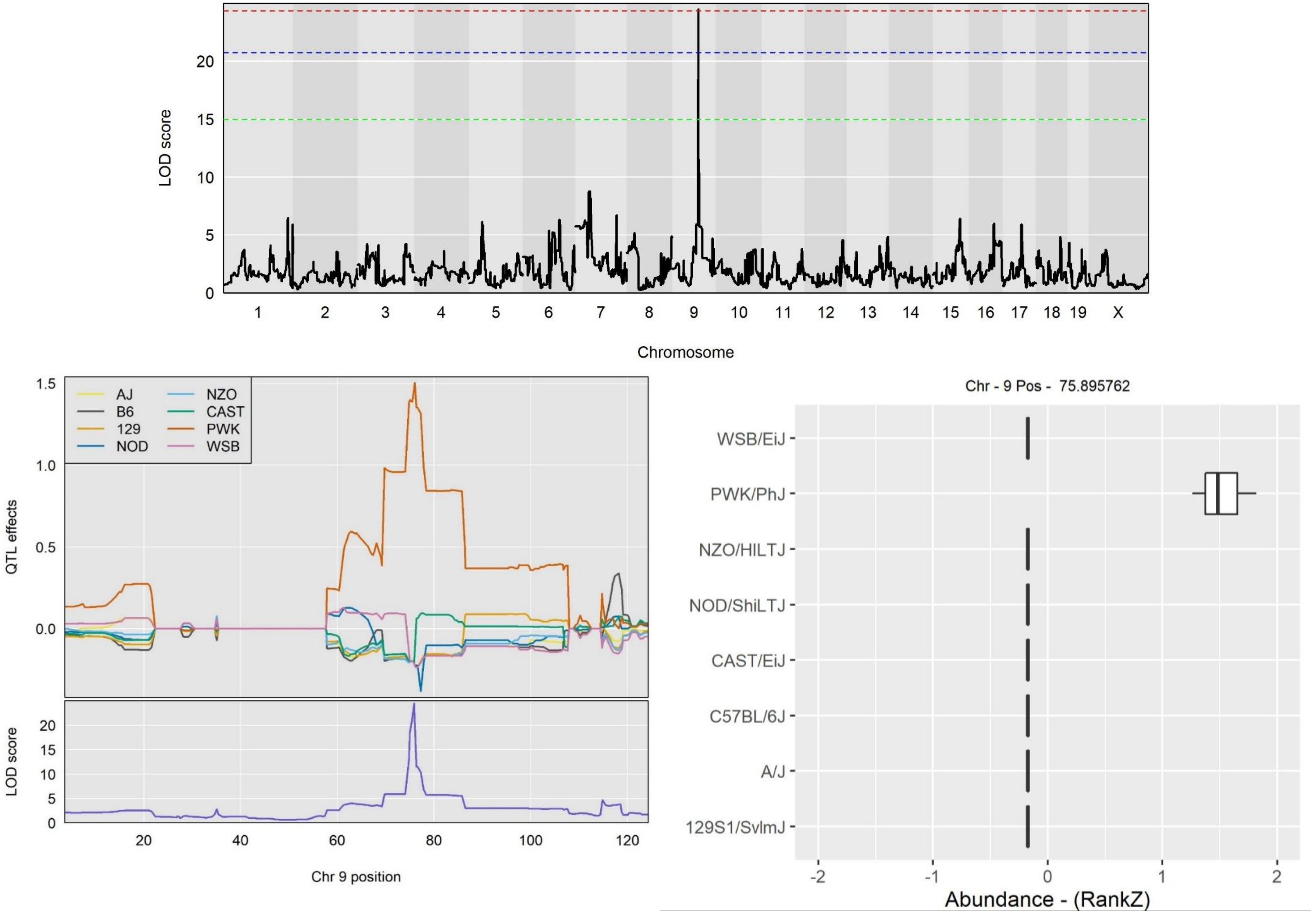

Fig Q9: Micabf9 - g_Erysipelotrichaceae

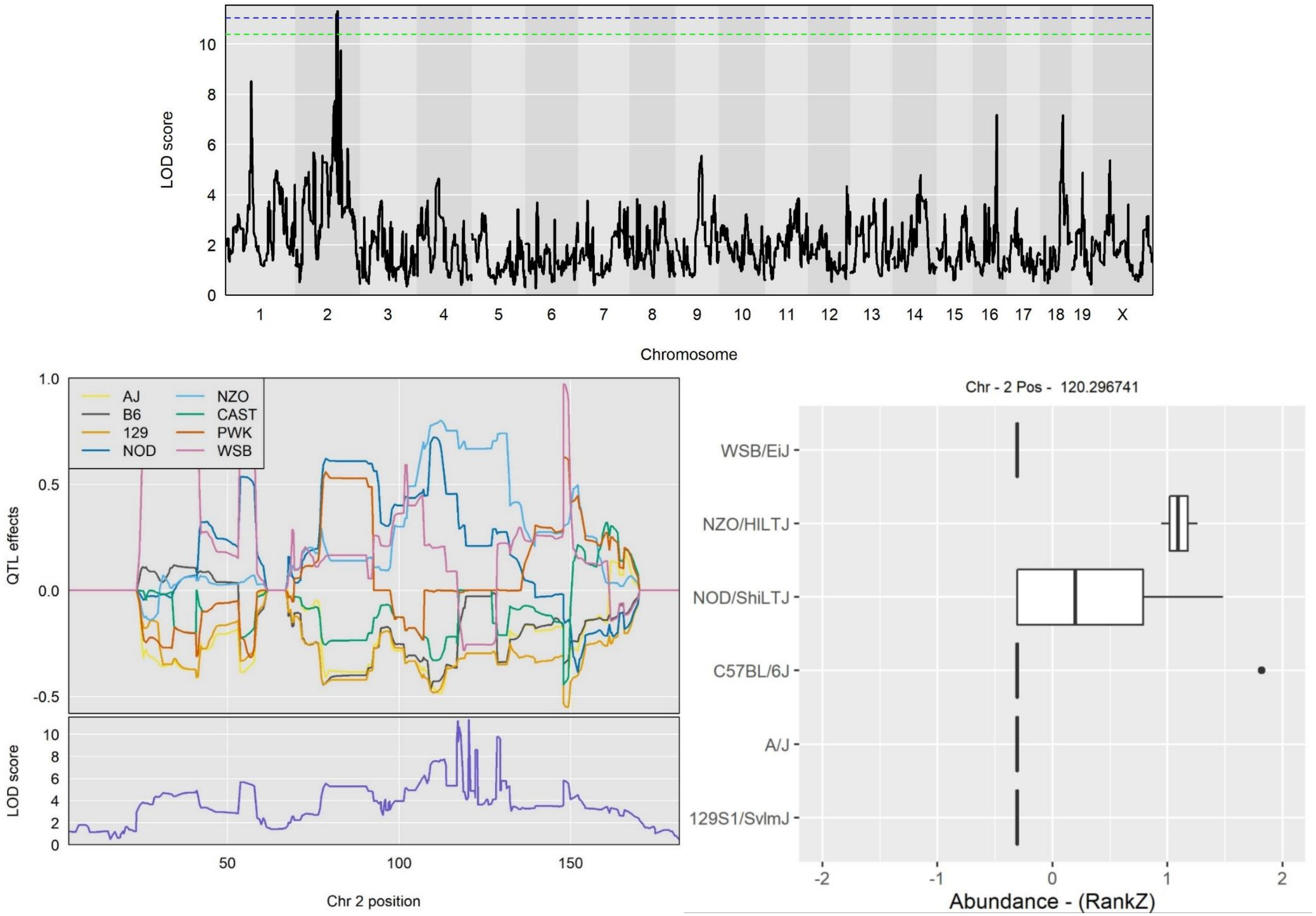

Fig Q10: Micabf10 - g_Candidatus_Arthromitus

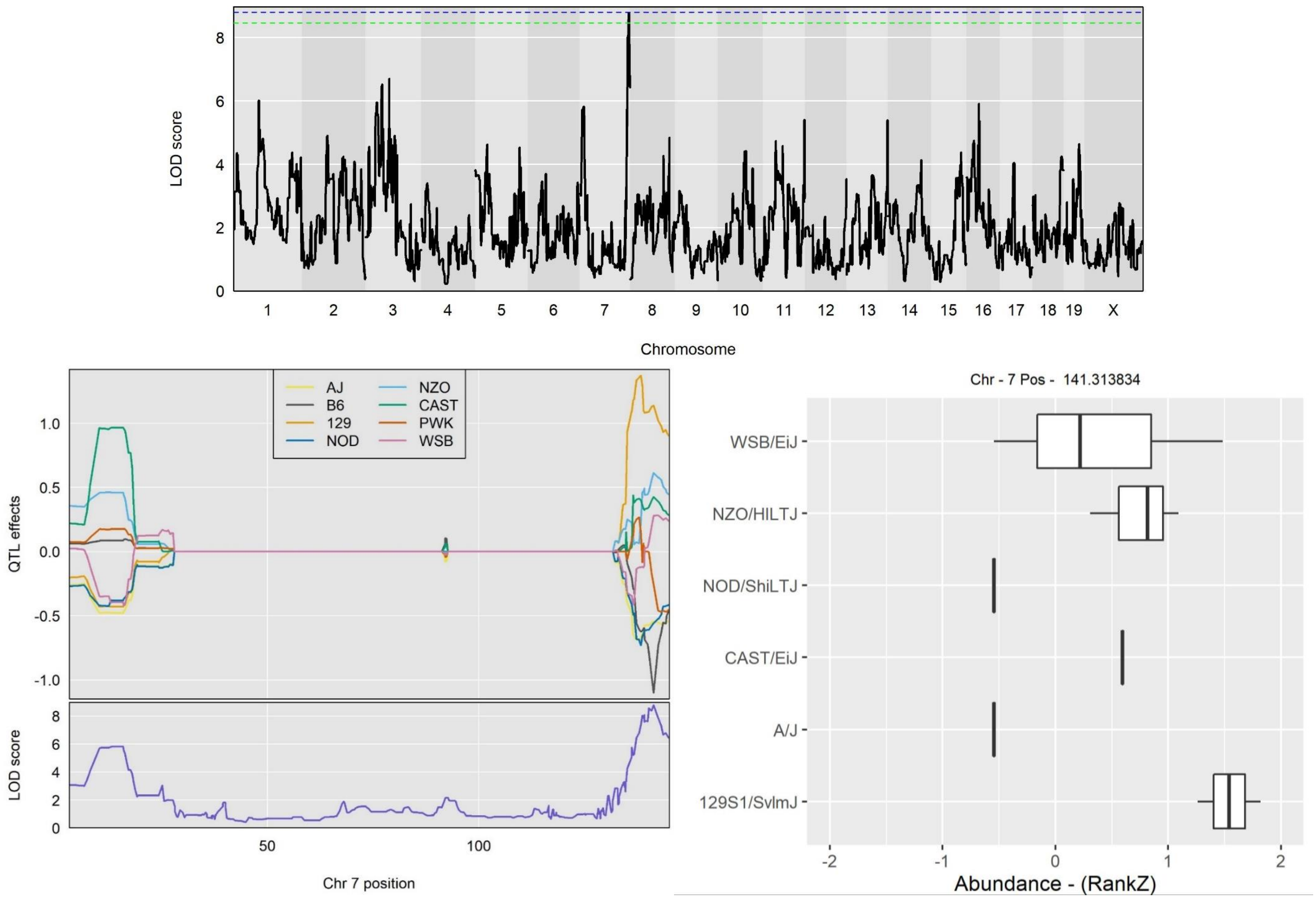

Fig Q11: Micabf11 - f_Lachnospiraceae / g_A2

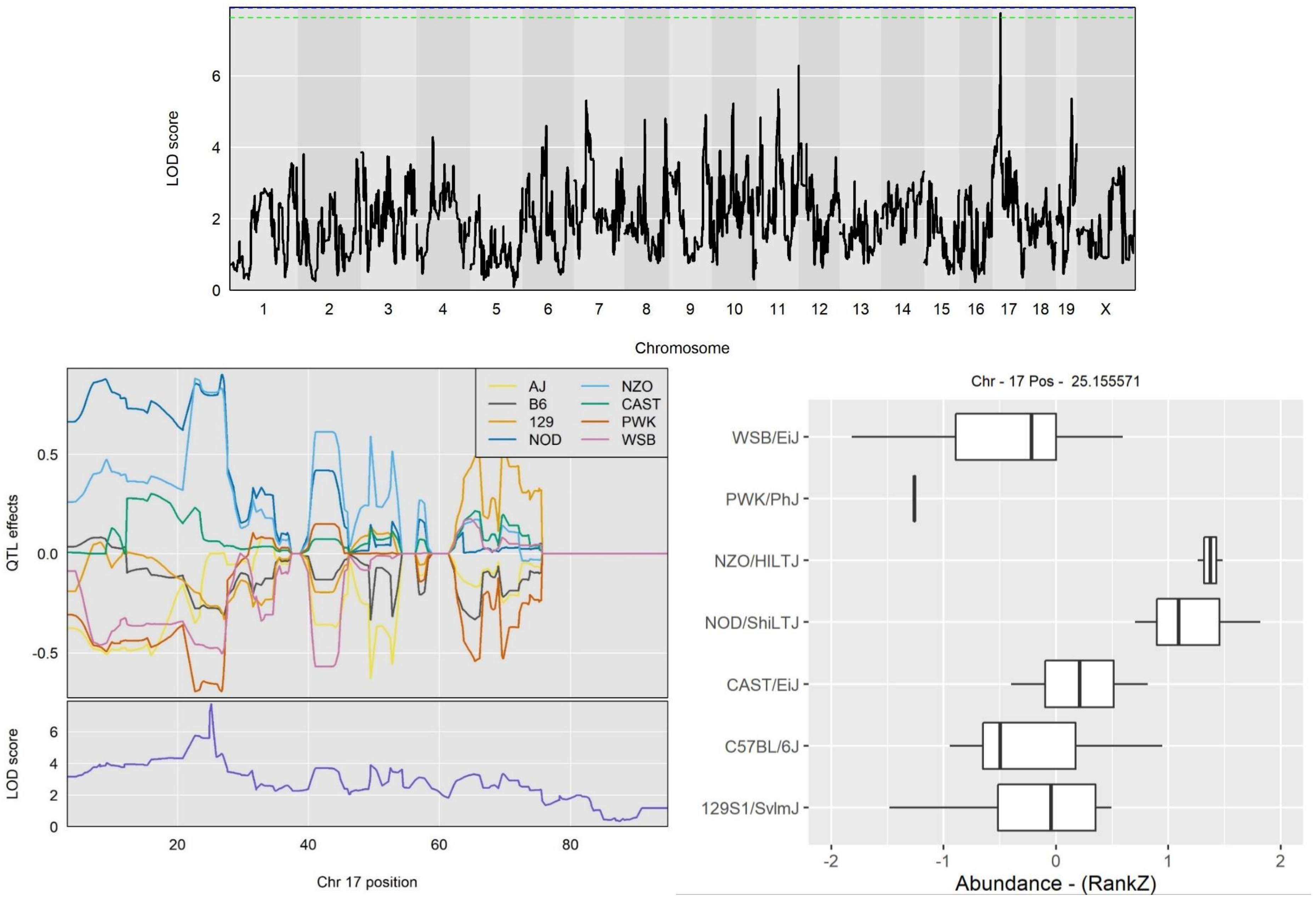

Fig Q12: Micabf12 - g_Lachnoclostridium

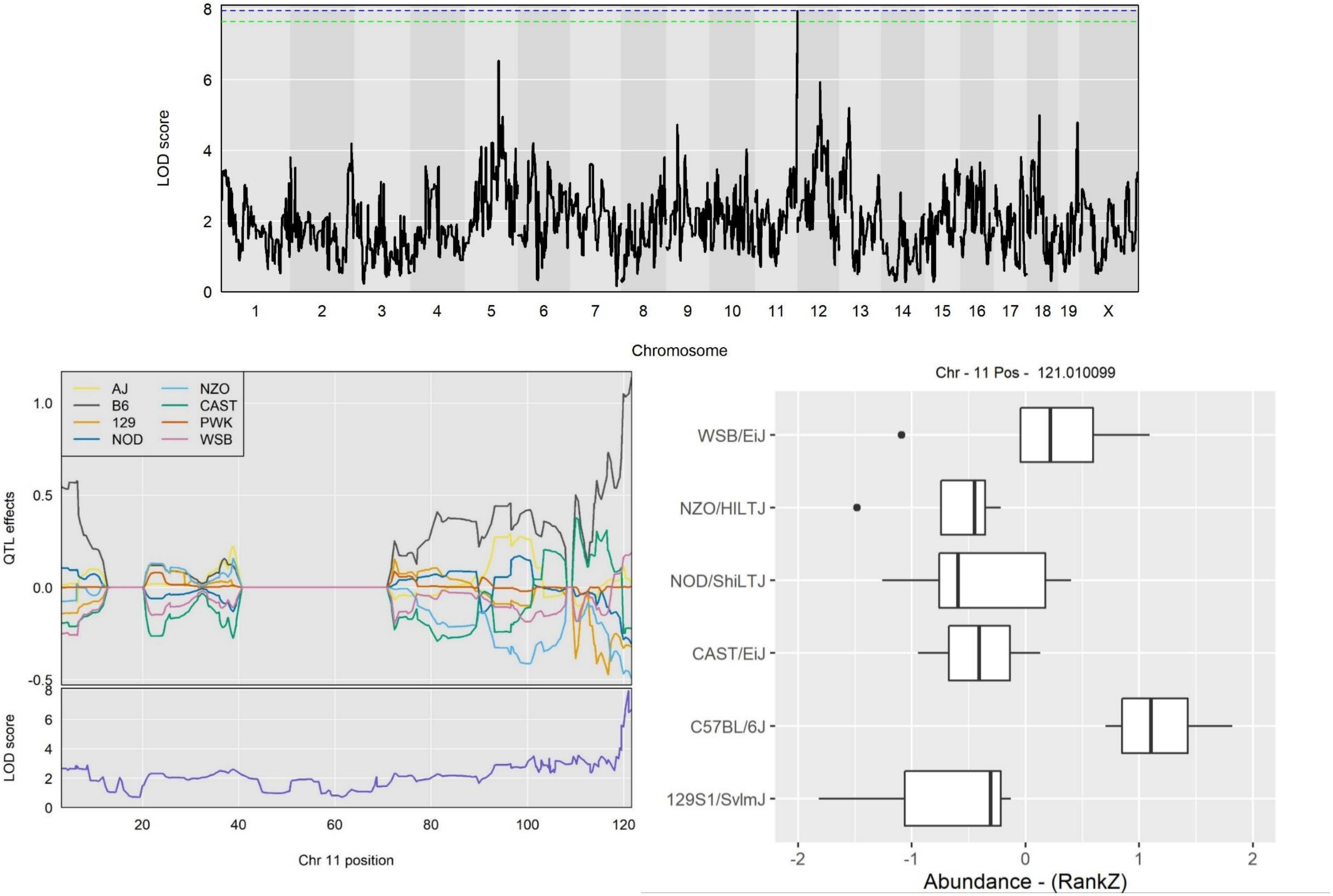

Fig Q13: Micabf13 - g_Lachnospiraceae_UCG006

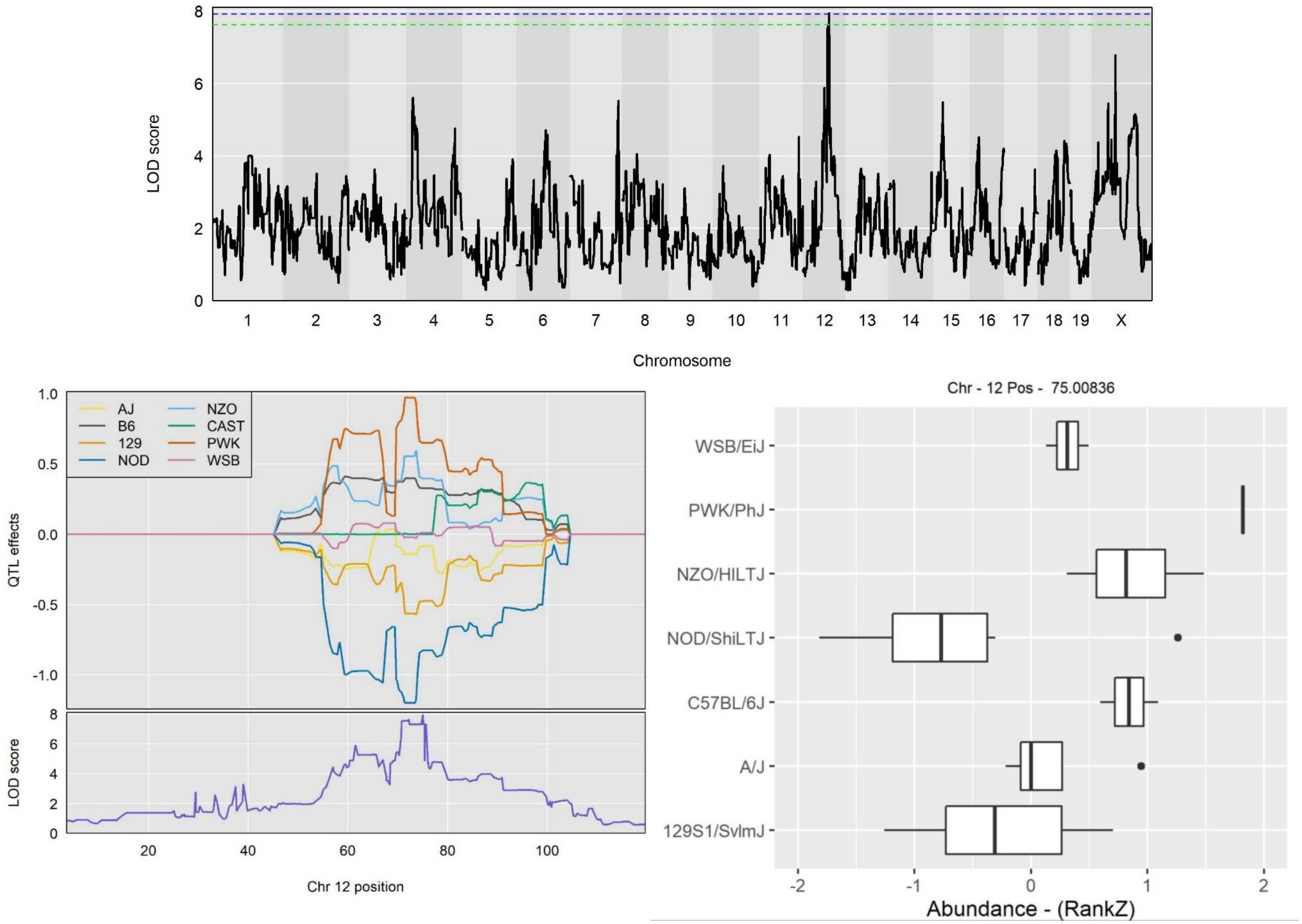

Fig Q14: Micabf14 - g_Ruminococcaceae

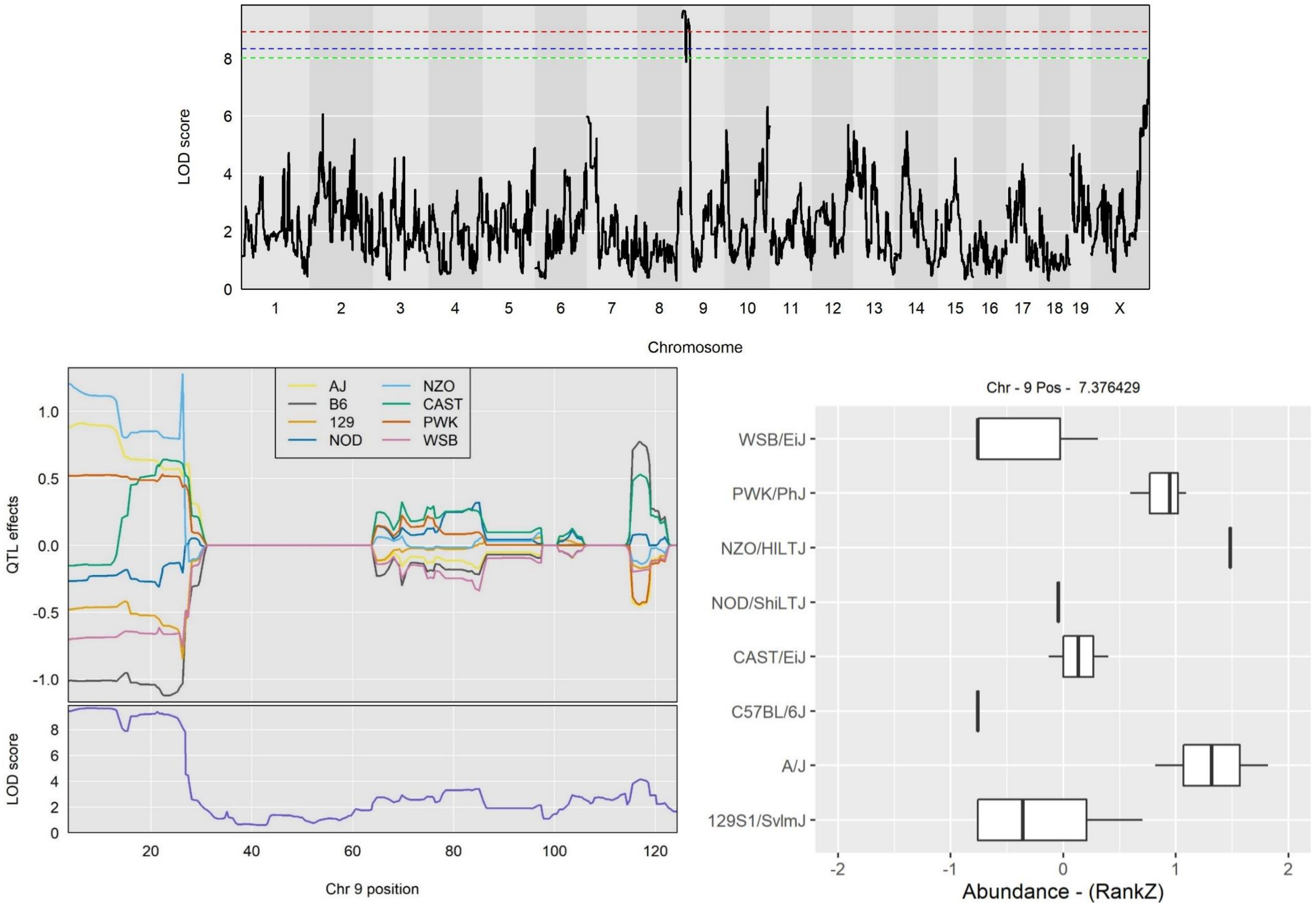

Fig Q15: Micabf15 - o_Oscillospirales / g_UCG10

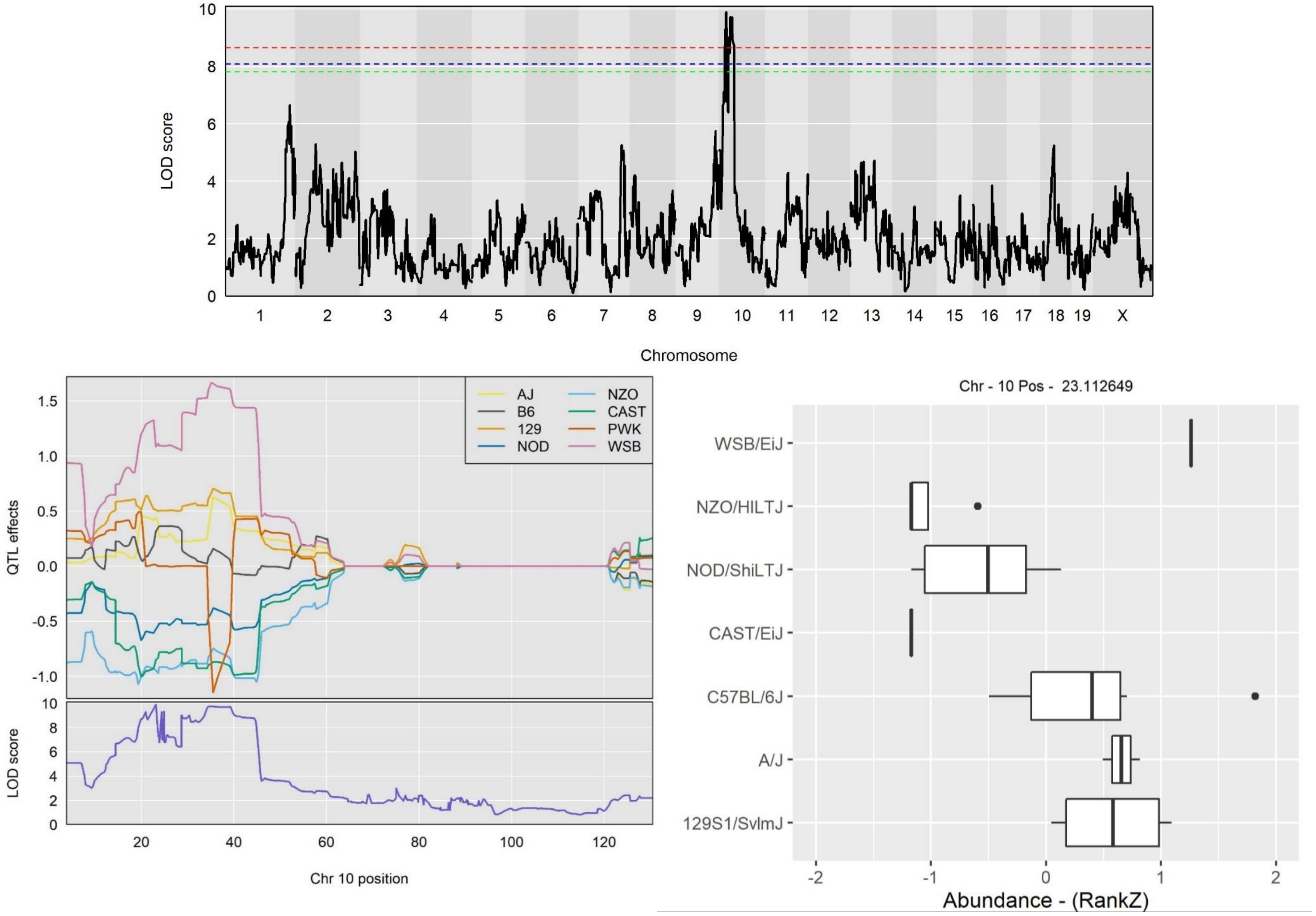

Fig Q16: Micabf16 - g_Peptococcus

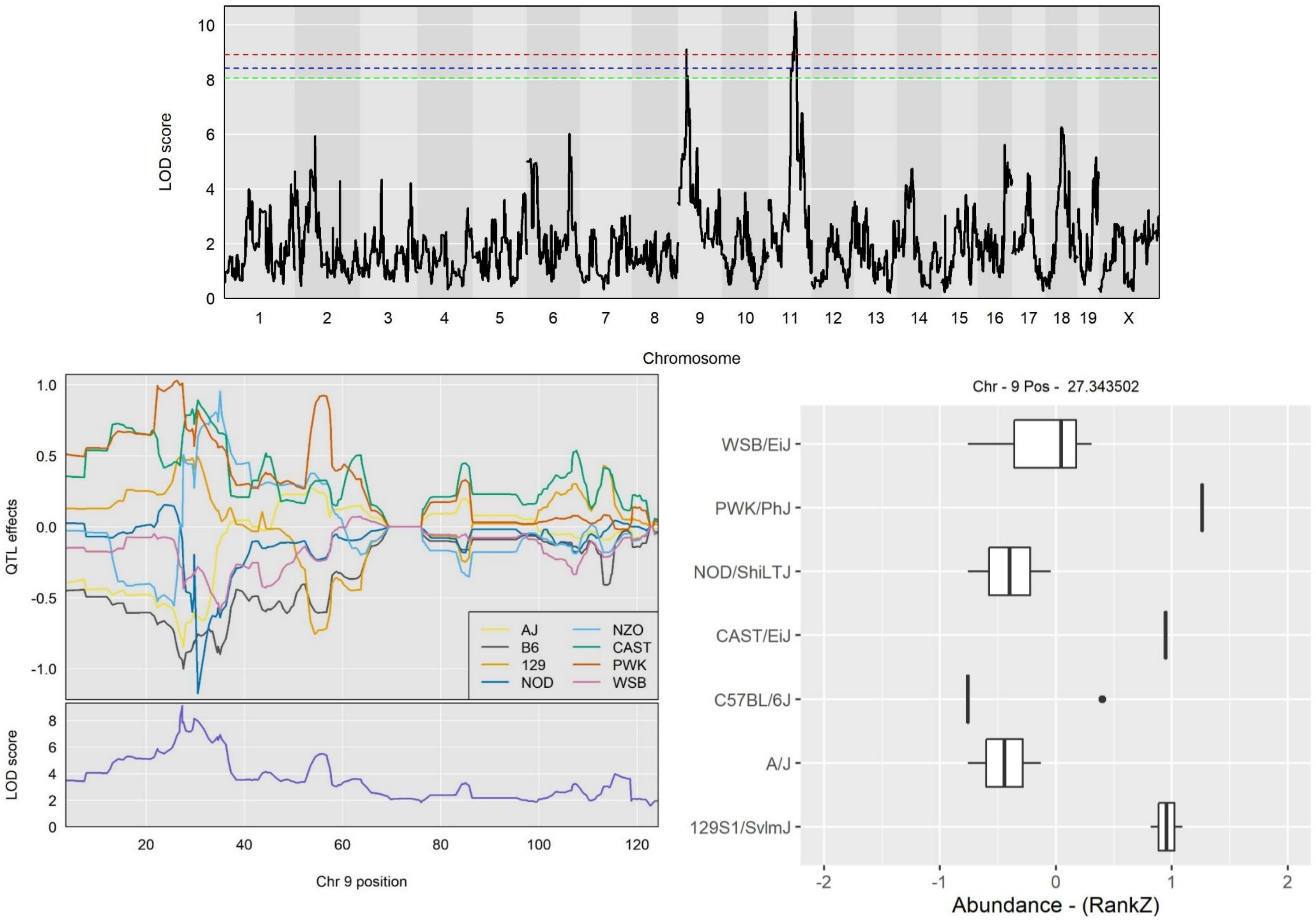

Fig Q17: Micabf17 - g_Peptococcus

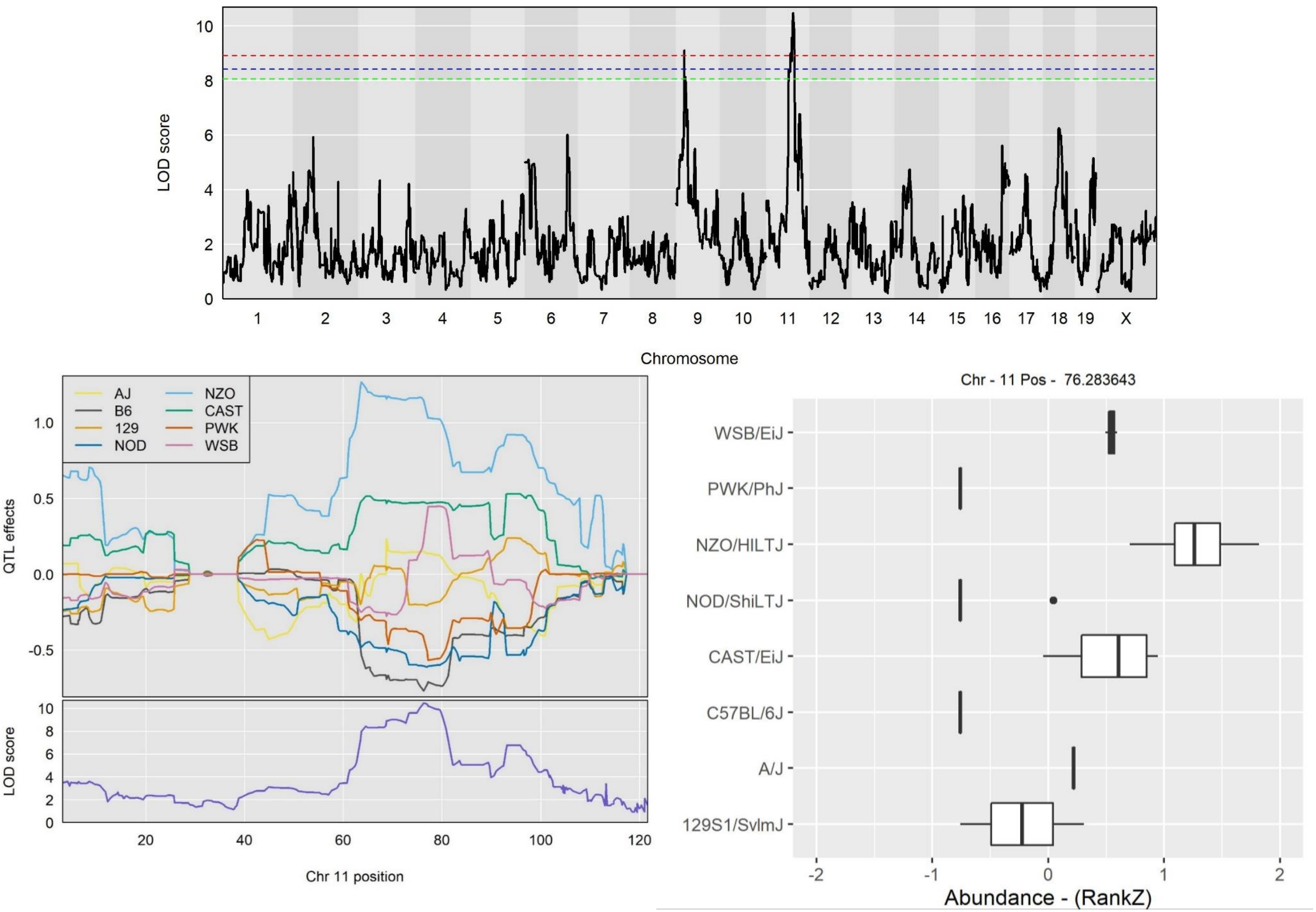

Supplement: Supplementary file 5 — Additional file 4: Figure S4. Plots for all the significant QTLs. A. LOD plot for rank transformed relative abundance values for significant genera. The dotted lines (Green – 85%, Blue – 90%, Red – 95%) represent significant thresholds calculated by 999 permutations. B. Founder effect plot and LOD plot for the significant chromosome. C. Genotype X Phenotype plot for the highest SNP on the significant chromosome. [file 40168_2023_1552_MOESM4_ESM.pdf]
